# Metabolomic and Cellular Mechanisms of Drug‐Induced Ototoxicity and Nephrotoxicity: Therapeutic Implications of Uric Acid Modulation

**DOI:** 10.1002/advs.202415041

**Published:** 2025-03-05

**Authors:** Suhan Guo, Cheng Cheng, Yunhao Wu, Kaidi Shen, Depeng Zhang, Bin Chen, Xinyu Wang, Luping Shen, Qixiang Zhang, Renjie Chai, Guangji Wang, Fang Zhou

**Affiliations:** ^1^ Key Laboratory of Drug Metabolism and Pharmacokinetics State Key Laboratory of Natural Medicines China Pharmaceutical University Nanjing 210009 China; ^2^ Department of Otolaryngology Head and Neck Surgery Nanjing Drum Tower Hospital Affiliated Hospital of Medical School Nanjing University Nanjing 210096 China; ^3^ Co‐Innovation Center of Neuroregeneration Nantong University Nantong 226001 China; ^4^ Medical Science and Technology Innovation Center Shandong First Medical University & Shandong Academy of Medical Sciences Jinan Shandong 250117 China; ^5^ State Key Laboratory of Digital Medical Engineering Department of Otolaryngology Head and Neck Surgery Zhongda Hospital School of Life Sciences and Technology Advanced Institute for Life and Health Jiangsu Province High‐Tech Key Laboratory for Bio‐Medical Research Southeast University Nanjing 210096 China; ^6^ Department of Pharmacy General Hospital of Ningxia Medical University Yinchuan 750004 China

**Keywords:** autophagy‐dependent ferroptosis, cisplatin, hearing loss, nephrotoxicity, stria vascularis, uric acid

## Abstract

Certain medications, including cisplatin and neomycin, often cause both hearing loss and renal dysfunction. This study aims to uncover the common mechanisms behind drug‐induced ototoxicity and nephrotoxicity to aid early diagnosis and treatment. Metabolomic analyses reveal simultaneous disruptions in endogenous metabolic networks in the kidney, inner ear, and serum after administrating cisplatin or neomycin. Notably, a marked elevation in uric acid (UA), a recognized indicator of renal tubular injury, is identified. Supplementing UA and inhibiting its renal excretion worsen hearing loss and hair cell damage. Single‐cell nucleus sequencing and immunohistochemistry reveal major changes in xanthine oxidase and *ABCG2*, crucial for UA metabolism, primarily in cochlear stria vascularis cells rather than hair cells. Cisplatin triggers a significant release of UA from stria vascularis cells, reaching concentrations sufficient to induce autophagy‐dependent ferroptosis in hair cells. In a coculture system, targeted interventions against these two proteins in stria vascularis cells, through either pharmacological inhibition or genetic manipulation, markedly decrease the elevated UA release and the subsequent ferroptosis of hair cells. These findings suggest a metabolic connection between the inner ear and the kidney, highlighting the therapeutic potential of modulating UA to mitigate drug‐induced nephrotoxicity and ototoxicity.

## Graphical abstract

Drug‐induced renal damage impairs uric acid excretion, causing its accumulation in the serum, which subsequently infiltrates the cochlea. Xanthine oxidase and the ABCG2 transporter, critical in uric acid metabolism and excretion, undergo pathological changes in renal tubular and cochlear stria vascularis cells. Targeted inhibition of specific genes in stria vascularis cells reduces uric acid levels, alleviating ferroptosis in cochlear hair cells.

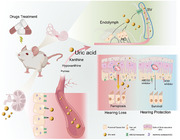

## 1 Introduction

Renal dysfunction and hearing loss are prevalent health issues that individually contribute to significant morbidity and often coexist.^[^ ^1^ ^]^ Despite the existence of profound developmental and physiological connections between hearing and renal functions, the associations between these two conditions remain largely unrecognized.^[^ ^2^ ^]^ Some widely used drugs, including platinum‐based chemotherapeutic agents like cisplatin (CDDP) and aminoglycoside antibiotics such as neomycin, exhibit dose‐dependent efficacy.^[^ ^3^, ^4^, ^5^ ^]^ However, their severe renal toxicity and permanent ear damage greatly limit their clinical use, thereby affecting therapeutic outcomes and patient prognosis.^[^ ^5^, ^6^ ^]^ Until recently, the US Food and Drug Administration (FDA) has not approved any therapies specifically for preventing hearing loss caused by ototoxic drugs.^[^ ^7^ ^]^ Although CDDP and aminoglycosides are fundamentally different molecules with unique physicochemical properties, the ototoxicity and nephrotoxicity they cause share many similarities, such as the destruction of cochlear sensory cells (both inner and outer hair cells), degeneration of the stria vascularis (SV), a significant reduction of spiral ganglion cells, and specific accumulation and damage to the epithelial cells of the proximal convoluted tubules of the kidney.^[^ ^8^, ^9^, ^10^ ^]^ However, the common material basis and precise mechanisms underlying drug‐induced renal dysfunction and hearing loss remain poorly elucidated.

Metabolomics has gained widespread application in recent years, particularly in the diagnosis and treatment of clinical diseases such as chronic kidney disease (CKD), as well as in evaluating surgical efficacy and prognosis.^[^ ^11^ ^]^ Our research demonstrated a correlation between aminoglycoside and CDDP toxicity and their accumulation in the kidney and inner ear,^[^ ^12^ ^]^ particularly in hair cells and tubular epithelial cells,^[^ ^13^ ^]^ while also showing that lipid metabolism disruptions exacerbate neomycin‐induced ear and kidney damage.^[^ ^14^ ^]^ Additionally, metabolite changes have also been reported to affect hearing.^[^ ^15^ ^]^ Studies have used metabolomics to investigate noise‐induced and sudden deafness,^[^ ^16^, ^17^ ^]^ as well as its applications and perspectives in age‐related hearing loss.^[^ ^18^ ^]^ Furthermore, Wan et al. detected alterations in purine metabolism within the cochlea of aged rats.^[^ ^19^ ^]^ The dramatic metabolic changes observed in vivo during drug‐induced ear and kidney injury inspired us to perform integrated metabolomics analysis of serum, kidney, and inner ear samples from model mice to unravel the underlying pathophysiological mechanisms.

Purine metabolism stands as a fundamental biological process crucial for sustaining life activities, and the regulation of purine metabolism plays a pivotal role in the treatment of various diseases.^[^ ^20^, ^21^, ^22^ ^]^ Uric acid (UA), the terminal product of purine metabolism, is primarily excreted by the kidneys. The relationship between elevated serum UA levels and kidney injury has long been a topic of interest for nephrologists.^[^ ^23^ ^]^ UA has been recognized as a marker of renal tubular injury,^[^ ^24^ ^]^ and both experimental models and clinical data have established a link between high UA levels and the development of CKD, as well as the progression of acute kidney injury.^[^ ^25^, ^26^, ^27^, ^28^ ^]^ Some studies have demonstrated that supraphysiological UA levels are also a risk factor for various metabolic disorders.^[^ ^29^ ^]^ Clinical reports indicate that patients suffering from gout face a 44% higher risk of hearing loss.^[^ ^30^ ^]^ Furthermore, disturbances in UA metabolism have been observed in patients with age‐related hearing loss.^[^ ^31^ ^]^ Additionally, UA levels exhibit a significant correlation with the severity of sudden deafness.^[^ ^32^ ^]^ However, previous studies have only demonstrated a correlation between UA and hearing, without providing direct evidence of its specific effects on cochlear hair cells. To date, the transport and metabolism of UA in the cochlea and its impact on hair cells have not been reported.

Several studies have explored the relationship between hearing impairment and kidney damage, however, the pathological connections between renal and cochlear functions have primarily been examined through small observational studies and case reports, lacking comprehensive analyses to fully elucidate their relationship. In this study, we performed a multiomics analysis on mouse models with drug‐induced auditory and renal injury, identifying UA as a critical factor in both directly and indirectly inducing damage to these organs. Furthermore, our investigation revealed that the modulation of UA metabolism and transport in SV can mitigate hearing impairment and ferroptosis of hair cells, thereby presenting a novel therapeutic approach for auditory recovery. This study contributes new insights into clinical treatment strategies and offers guidance for the rational regulation of serum UA levels in drug‐induced ototoxicity and nephrotoxicity.

## 2 Results

### 2.1 Coalteration of Purine Metabolism Pathway in the Serum, Kidneys, and Inner Ears in Drug‐Induced Nephrotoxicity and Ototoxicity Mouse Models

**Figure 1:**
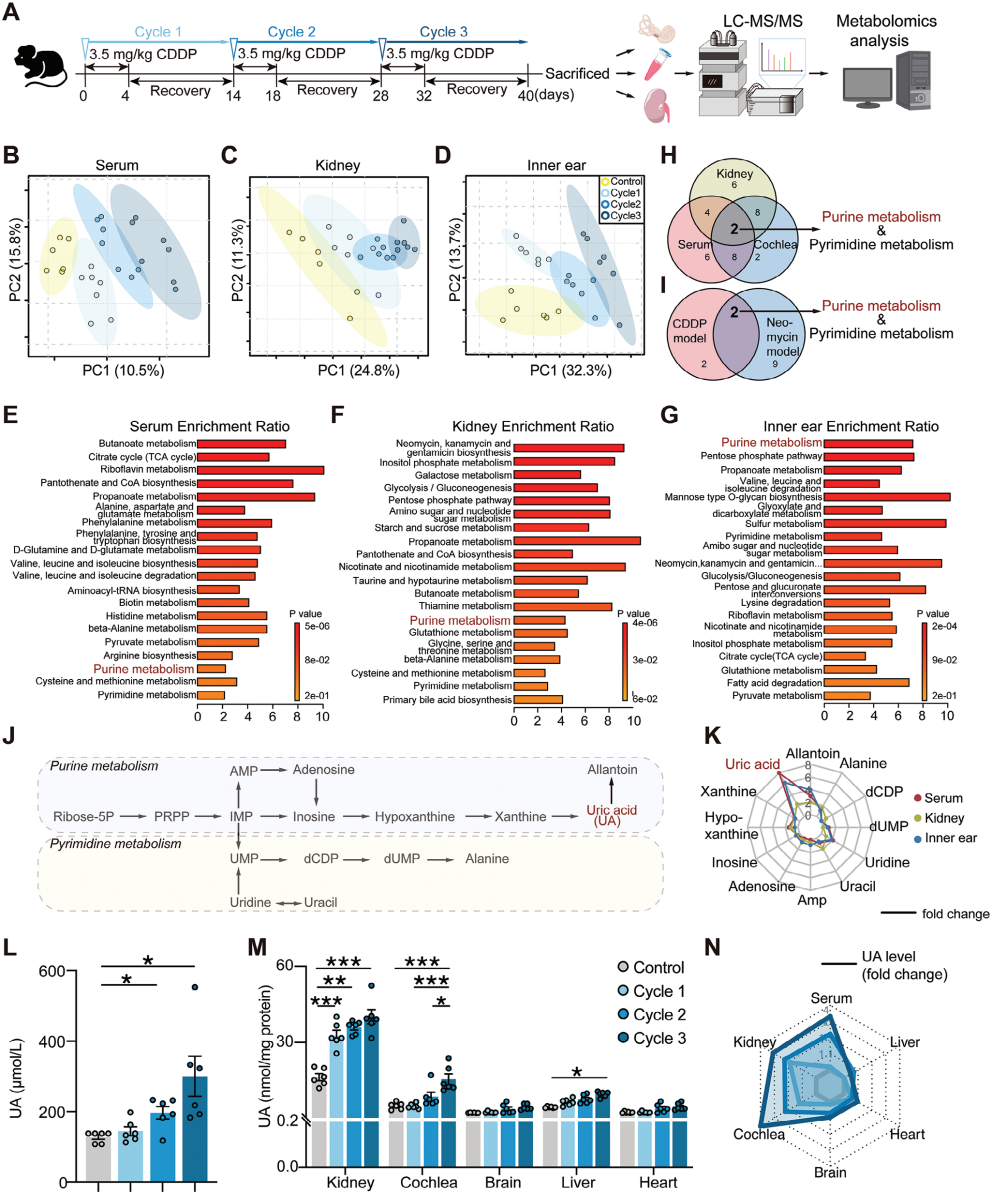
Metabolomics analysis of mice serum, kidney, and inner ear after CDDP treatment. A) Overview of sample collection and experimental design. B–D) Principal component (PC) analysis in serum, kidney, and inner ear from the studied groups, respectively (*n* = 6–7). E–G) Kyoto Encyclopedia of Genes and Genomes (KEGG) enrichment analysis, based on differential metabolites from studied groups in serum, kidney, and inner ear after CDDP treatment, identified distinct metabolic pathways. H,I) Venn diagrams illustrating commonalities and differences in metabolic pathways between serum, kidney, and inner ear after CDDP treatment, and between two drug‐induced mouse models. J) Schematic illustration of detectable metabolites in the purine and pyrimidine metabolic pathways. K) Radar plot showing changes in metabolites in serum, kidney, and inner ear after three cycles of CDDP treatment. L,M) Quantitative detection of UA levels in serum and various tissues in mice over three cycles of CDDP treatment by UA assay kit (*n* = 6). N) Radar plot showing the relative fold change in UA in various tissues in mice after the third cycle of CDDP treatment by UA assay kit. Data are expressed as means ± SEM. Statistical analysis: one‐way ANOVA for (L, M). *p < 0.05, **p < 0.01, ***p < 0.001. CDDP, cisplatin; UA, uric acid.

To identify common metabolic features and shared material bases between drug‐induced ear and kidney injuries, CDDP and neomycin (Figure S2A, Supporting Information) were used to establish drug‐induced hearing and kidney injury models, respectively. Renal injury was confirmed by histopathological staining (Figures S1A and S2D, Supporting Information), serum creatinine (Scr) levels (Figures S1B and S2C, Supporting Information), and blood urea nitrogen (BUN) levels (Figures S1C and S2B, Supporting Information), while ototoxicity was assessed using auditory brainstem response (ABR) measurements (Figures S1D and S2E, Supporting Information) and histopathological staining of hair cells (HCs) and the SV (Figures S1E–H and S2F–I, Supporting Information). In the model of ear–kidney coinjury, untargeted metabolomics analysis using liquid chromatography–mass spectrometry/MS (LC–MS/MS) (**Figure** **1**A) revealed disruptions in endogenous metabolic networks in serum, inner ear, and kidney samples from CDDP‐treated mice. Principal component analysis showed distinct boundaries and time‐dependent separation of metabolites in the CDDP group compared to the control group, indicating significant differences in cumulative metabolites (Figure 1B–D). Subsequent enrichment analysis of metabolic pathways identified purine metabolism as the most significantly altered pathway in the inner ear and a highly enriched pathway in serum and kidney samples (Figure 1E–G). A similar trend was observed in mice with neomycin‐induced ear and kidney injuries (Figure S2J, Supporting Information), suggesting a prominent role of purine metabolism in drug‐induced ear and kidney dysfunction.

Venn diagrams illustrated two common metabolic pathways, purine metabolism, and pyrimidine metabolism, among the three types of samples in CDDP‐treated mice (Figure 1H) as well as in the neomycin‐induced model (Figure 1I). A semiquantitative study of ten relevant metabolites detectable in both pathways was conducted to identify key metabolites (Figure 1J). Results showed a substantial elevation of the purine metabolite UA in all samples from serum, kidney, and inner ear after CDDP administration (Figure 1K). Further quantitative analysis indicated that UA gradually accumulated in the kidney and cochlea with prolonged administration time, while other organs such as the liver, brain, and heart were less affected or not significantly affected (Figure 1L,N). Notably, UA levels in the kidney increased significantly after one cycle of CDDP administration, whereas the elevation of UA in the cochlea occurred later than the changes in the kidneys. Similar results were observed in neomycin‐induced ear and kidney injury models (Figure S2N,O, Supporting Information). These findings suggest that UA can sensitively reflect renal function, and its accumulation in the cochlea compared to other organs may have an inherent mechanism.

### 2.2 Supplementing Uric Acid and Inhibiting Its Renal Excretion Exacerbate Hearing Loss and Renal Dysfunction

**Figure 2:**
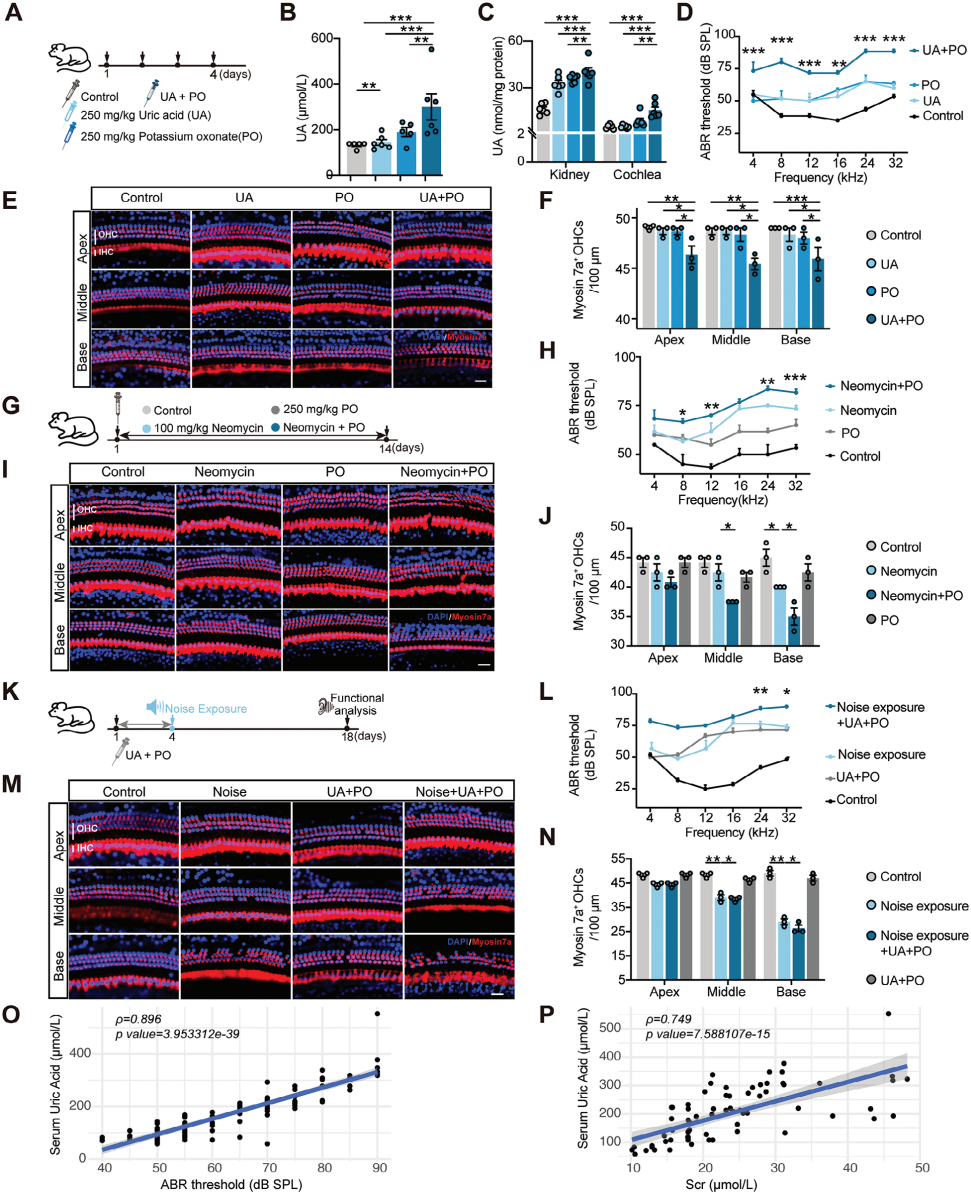
Elevated uric acid levels exacerbate hearing loss. A) Schematic diagram of the experiment to establish different levels of UA in mice. B,C) Quantitative detection of UA levels in serum, kidney, and cochlea from the studied groups (*n* = 5–6). D) ABR thresholds in each group (*n* = 3). E) Representative confocal images of hair cells stained with Myosin 7a (red) and DAPI (blue) in mice cochleae for each group, scale bar: 20 µm. F) Myosin 7a^+^ OHC quantification in mice for each group (*n* = 3). G) Schematic diagram of the experiment to establish ear and kidney injury caused by neomycin aggravated by high UA in vivo in mice. H) ABR thresholds in each group (*n* = 3). The statistical differences were analyzed between neomycin + PO group and neomycin group. I) Representative confocal images of hair cells labeled with Myosin 7a (red) and DAPI (blue) in mice cochleae for each group, scale bar: 20 µm. J) Myosin 7a^+^ OHC quantification in mice for each group (*n* = 3). K) Schematic diagram of the experiment to establish a noise exposure model under high UA levels in mice. L) ABR thresholds in each group (*n* = 3). The statistical differences were analyzed between noise exposure + UA + PO group and noise exposure group. M) Representative confocal images of hair cells labeled with Myosin 7a (red) and DAPI (blue) in mice cochleae for each group, scale bar: 20 µm. N) Myosin 7a^+^ OHC quantification in mice for each group (*n* = 3). O) Scatterplot depicting the correlation between in vivo UA levels and hearing function (ABR threshold at 32 kHz) in the aforementioned mouse model. P) Scatterplot depicting the correlation between UA levels and kidney function (Scr levels) in the aforementioned mouse model in vivo. Data are expressed as means ± SEM. Statistical analysis: spearman rank correlation test for (O, P); one‐way ANOVA for (B, C, F, J, N); two‐way ANOVA for (D, H, L). *p < 0.05, **p < 0.01, ***p < 0.001. UA, uric acid; PO, potassium oxonate; Scr, serum creatinine; BUN, blood urea nitrogen; OHC, outer hair cell; IHC, inner hair cell; ABR, auditory brainstem response.

Chronically high level of UA in circulation is a recognized risk factor for kidney injury,^[^ ^33^ ^]^ and it has been reported a correlation with the severity of sudden deafness.^[^ ^32^ ^]^ However, the relationship between UA and drug‐induced ear damage has rarely been reported. To explore the effects of UA on hearing function, different mouse models were established to mimic different pathologic circumstances. First, a mouse model with elevated UA levels was established by exogenous supplementation of UA (250 mg kg^−1^ UA, intraperitoneally), inhibition of UA excretion (250 mg kg^−1^ potassium oxonate, PO, intragastrically), and a combination of both approaches. The combination treatment group displayed a 2.3‐fold increase of UA in serum, and significant increases in kidney and cochlea (**Figure** **2**A–C). With the increased levels of UA, the ABR thresholds of the single treatment groups were higher than that of the control group, while the combination treatment group showed a significant increase in ABR thresholds at all the frequencies detected (Figure 2D). Similarly, immunofluorescence staining for the hair cell marker Myosin 7a indicated significant hair cell loss in mice with combined treatment compared to single treatment (Figure 2E,F). Pathophysiological changes of kidneys, Scr, and BUN showed more severe impairment of renal structure and function in the combination treatment group (Figure S3A–D, Supporting Information). These findings suggest the direct action of UA on the function of the inner ear and kidneys.

Second, the effect of UA on drug‐induced ototoxicity and nephrotoxicity was further investigated. In the neomycin‐induced ear and kidney injury model, inhibition of UA excretion with PO increased the neomycin‐induced upregulation of ABR threshold and exacerbated the loss of hair cells (Figure 2G–J), accompanied by significant elevation of UA in serum, ear, and kidney tissues (Figure S3H,I, Supporting Information). Neomycin‐induced renal dysfunction was also exacerbated by abnormal UA levels (Figure S3E–G, Supporting Information).

Then, to better assess the effects of UA on a distinct organ—the ear—we used a noise exposure model (Figure 2K). Noise‐injured mice with elevated UA levels (treated with UA and PO) exhibited greater hearing impairment and hair cell loss than the noise exposure group (Figure 2L–N). These results suggest that abnormal elevation of blood UA in vivo is often accompanied by severe renal failure and hearing loss. Further correlation analysis shows that serum UA levels are positively related to ABR threshold and blood creatinine levels (Figure 2O,P), highlighting the important role of UA in ear and renal function.

### 2.3 Uric Acid Directly Damages Cochlear Explants and Exacerbates Drug‐Induced Loss of Hair Cells

**Figure 3:**
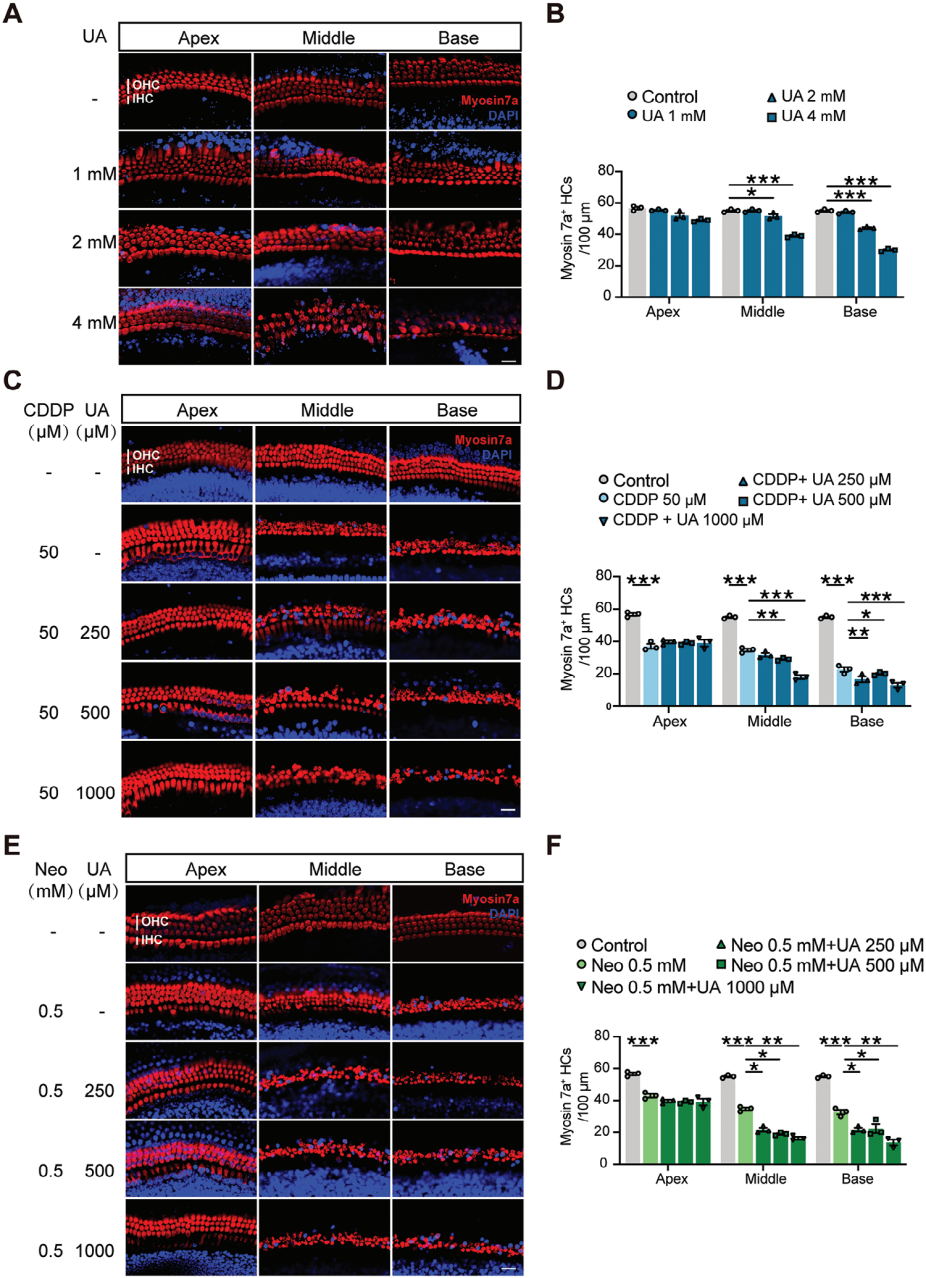
Uric acid directly damages cochlear explants and exacerbates drug‐induced loss of hair cells. A) Immunofluorescence analysis of hair cells (HCs) with or without UA at different concentrations for 24 h. Myosin 7a positive HCs are shown in red, and nuclei are labeled in blue. Scale bar: 20 µm. B) Statistical analysis of HCs in each group (*n* = 3). C) Effects of different levels of UA on HCs in the apical, middle, and basal turns after CDDP treatment for 24 h. Scale bar: 20 µm. D) Statistical analysis of HCs in each group (*n* = 3). E) Effects of different levels of UA on HCs in the apical, middle, and basal turns after neomycin treatment for 24 h. Scale bar: 20 µm. F) Statistical analysis of HCs in each group (*n* = 3). Data are expressed as means ± SEM. *p < 0.05, **p < 0.01, ***p < 0.001 by one‐way ANOVA. Neo, neomycin.

To assess the direct impact of UA on hair cells, cochlear explants from P3 wild‐type C57BL/6 mice were exposed to varying concentrations of UA (0, 1, 2, and 4 mm) for 24 h. Myosin 7a staining showed orderly arranged hair cells with good cellular integrity in the control group. However, in groups exposed to high concentrations of UA, there was significant hair cell loss, disrupted arrangement, and abnormal cell morphology, especially in the middle and basal turns of the cochlea. These changes exhibited a marked dose‐ and time‐dependent effect (**Figures** **3**A,B and S4E,F (Supporting Information)). To explore the indirect role of UA in drug‐induced ototoxicity and nephrotoxicity, a Cell Counting Kit‐8 (CCK‐8) assay was used to evaluate cytotoxicity in renal tubular epithelial cells (TCMK‐1) and cochlear hair cells House Ear Institute‐Organ of Corti 1 (HEI‐OC1). The findings indicated that UA stimulation exacerbated drug‐induced cytotoxicity in a dose‐dependent manner (Figure S4A–D, Supporting Information). To confirm whether UA similarly aggravates CDDP‐ or neomycin‐induced cochlear explant hair cell injury, the surviving hair cells in each group were quantified by immunostaining with Myosin 7a. Representative confocal images showed a significant decrease in hair cell count, particularly in the mid‐ and basal‐turns, after exposure to CDDP or neomycin alone. This loss was further exacerbated by UA coincubation at concentrations of 250 or 500 µm (Figure 3C–F), which are significantly lower than the toxic concentration of UA alone (Figure 3A). This damage initially affected the basal turn and progressively spread to the mid‐turn, demonstrating a clear dose‐dependent effect.

### 2.4 The Abnormal Uric Acid Metabolism Is Closely Linked to the Special Alteration of *ABCG2* and Xanthine Oxidase in the Kidney and Cochlea

**Figure 4:**
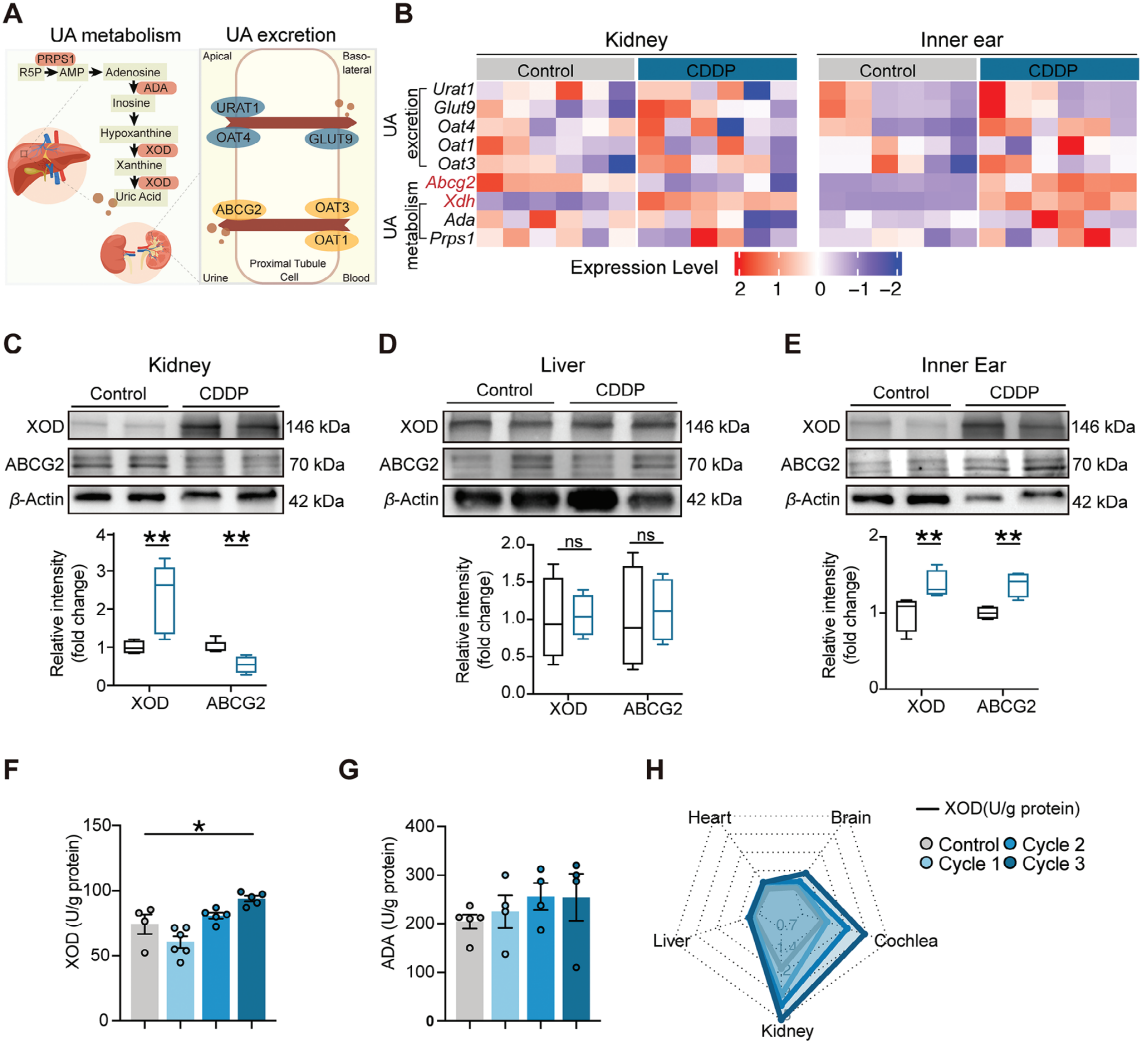
Altered uric acid transporters and metabolic enzymes in the kidney and cochlea after drug administration. A) Diagram illustrating the transporters and biological processes involved in UA metabolism and excretion. B) Heatmap depicting changes in mRNA expression of key factors regulating UA levels in mouse inner ear and kidney tissues following CDDP administration (*n* = 6). C–E) Western blot analysis of XOD and *ABCG2* protein levels in the kidney, liver, and inner ear after CDDP administration (*n* = 3). F,G) Serum activities of the rate‐limiting enzymes of purine metabolism, XOD and ADA, over three cycles of CDDP treatment (*n* = 4–5). H) Radar plot showing the activity of XOD in various organs following CDDP administration. Data are expressed as means ± SEM. Statistical analysis: Student's *t* test for (C, D, E, I); one‐way ANOVA for (F). *p < 0.05, **p < 0.01, ***p < 0.001. ADA, adenosine deaminase.

Elevated UA levels in the body may result from both excessive production and compromised excretion.^[^ ^34^ ^]^ As depicted in **Figure** **4**A, UA is the final product of purine metabolism through a two‐step catalytic process involving xanthine oxidase (XOD), the rate‐limiting enzyme of UA metabolism. Its excretion in renal tubular cells occurs either into the peripheral blood via a reabsorption transporter or out of the body with urine via a secretion transporter.^[^ ^35^, ^36^ ^]^ Despite the widespread distribution of enzymes and transporters involved in UA‐related biological processes throughout the body, their role in the inner ear remains poorly documented. In this study, we delved into the reasons behind the overproduction of UA, considering both metabolism and excretion. First, we assessed the mRNA expression levels of crucial elements that govern UA biological processes and discovered that the UA transporter *ABCG2* was notably downregulated in the mouse kidney after CDDP administration, but significantly upregulated in the inner ear. Moreover, *Xdh* (the gene name of XOD) exhibited a substantial upregulation in both the inner ear and kidney (Figure 4B). Western blot analysis confirmed the significant modifications in the protein levels of *ABCG2* and XOD in the inner ear as well as the kidney after drug‐induced injury when compared to liver tissue (Figure 4C–E). XOD activity underwent substantial changes after drug administration in serum (Figure 4F), ear, and kidney rather than other tissues (Figure 4H). By contrast, another rate‐limiting enzyme adenosine deaminase did not alter significantly after drug administration (Figure 4G). These results suggest that the abnormal elevation of UA may be due to changes in *ABCG2* and XOD.

### 2.5 Drug‐Induced Alterations of *ABCG2* and Xanthine Oxidase in the Inner Ear Predominantly Occur in the Stria Vascularis

**Figure 5:**
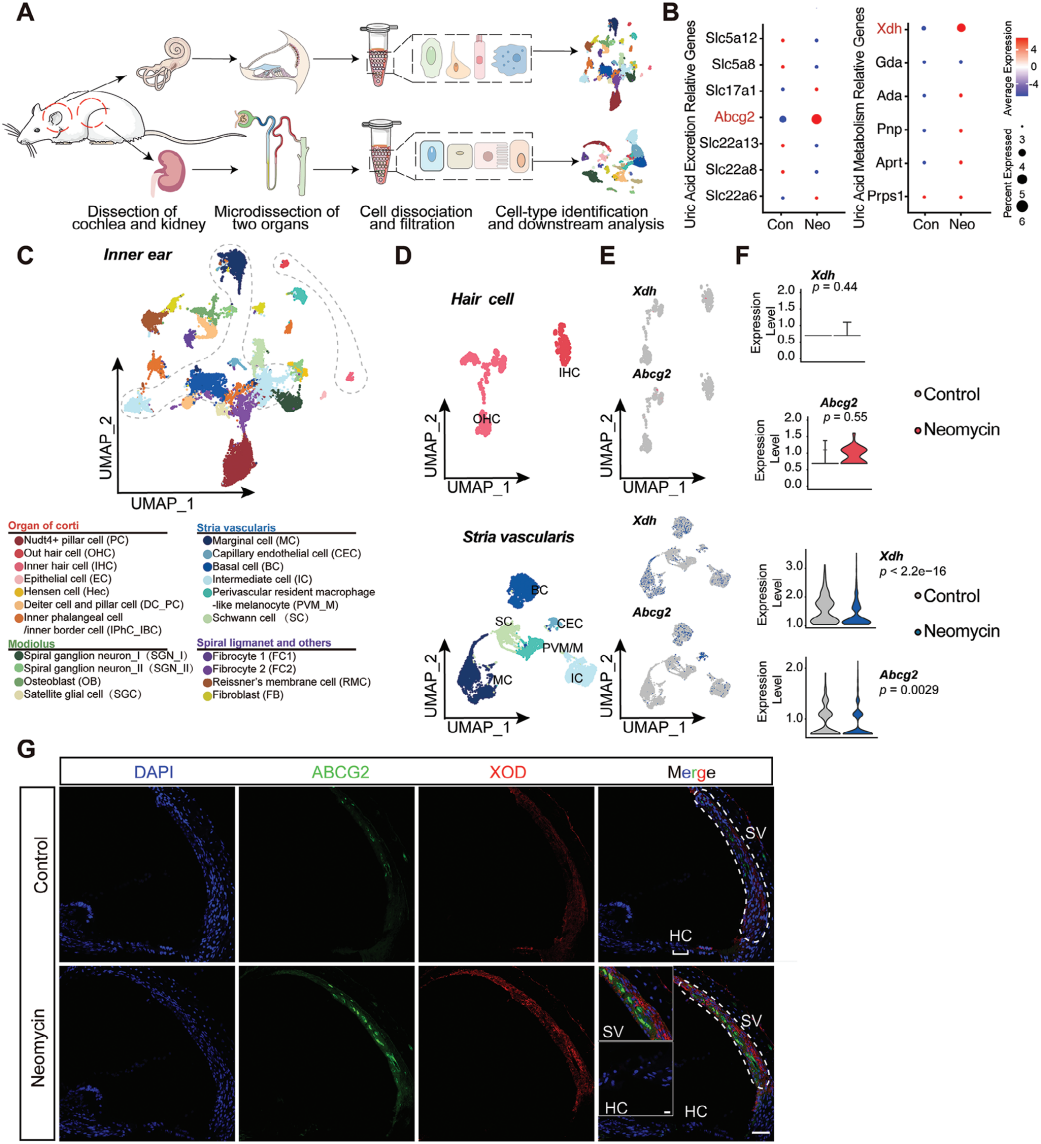
Establishment of single‐cell transcriptome landscape of mouse cochlea and kidney after neomycin administration. A) Diagram depicting the procedure for establishing a neomycin‐induced ototoxicity and nephrotoxicity damage model using single‐cell RNA sequencing (snRNA‐seq), along with subsequent verification of molecular mechanisms. B) Dot plot showing changes in genes associated with UA metabolism in the inner ear after neomycin administration. C) UMAP plot showing the distribution of different cell types in the cochlea. D) UMAP plot showing the subset of the stria vascularis composed of MC, BC, IC, CEC, SC, and PVM/M. UMAP plot showing the subset of hair cell composed of IHC and OHC. E) UMAP plot displaying the distribution of *Xdh* and *ABCG2* genes in the SV subset and hair cell subset. F) Violin plots of selected gene expression in stria vascularis cells and hair cells after neomycin administration. G) Representative immunohistochemical images of XOD (red) and *ABCG2* (green) in the mouse cochlea cross‐section after neomycin administration. Scale bar: 20 µm.

To pinpoint the specific cell subpopulations within the ear and kidney tissues that play a pivotal role in the biological process of UA dysregulation in the disease model, we constructed mouse cochlear and kidney single‐cell RNA sequencing (snRNA‐seq) profiles following drug administration (**Figure** **5**A). After eliminating low‐quality cells, performing quality control, and removing cyclic, mitochondrial, and bicellular genes, we obtained a total of 28 599 cells in the cochlear dataset for subsequent analysis. Bubble plot results indicated notably elevated levels of the *ABCG2* and *Xdh* genes compared to other UA transporters or purine‐metabolizing enzymes, with these changes being more pronounced after drug exposure (Figure 5B). By comparing transcriptional profiles with established cell‐type‐specific markers, we identified 21 major cell types in cochlear tissue, including cells around the Corti, Mosiolus, SV, and spiral ligament (Figure 5C and Figure S5A (Supporting Information)). Further analysis and uniform manifold approximation and projection (UMAP) plots showed that the two genes, *ABCG2* and *Xdh*, were primarily distributed in the SV of the cochlea, while they were expressed at very low levels in outer or inner hair cells (Figure 5D,E). *Xdh* was predominantly expressed in basal cells and marginal cells, whereas *ABCG2* was mainly localized in capillary endothelial cells and basal cells. Violin plots derived from the snRNA‐seq dataset revealed no significant changes in *ABCG2* and *Xdh* expressions within hair cells, but a significant upregulation was observed in the SV subpopulation after drug administration (Figure 5F). The immunofluorescence on the frozen section further confirmed the expression patterns of *ABCG2* and XOD identified by snRNA‐seq analysis. XOD (red) exhibited high expression levels in the marginal and basal cell layers of the SV, while *ABCG2* (green) was expressed in the capillary endothelial region (Figure 5F,G). They were both induced dramatically after neomycin treatment in SV rather than hair cells.

### 2.6 CDDP Induces a Notable Release of Uric Acid from Stria Vascularis Cells and Subsequently Triggers Autophagy‐Dependent Ferroptosis of Hair Cells

**Figure 6:**
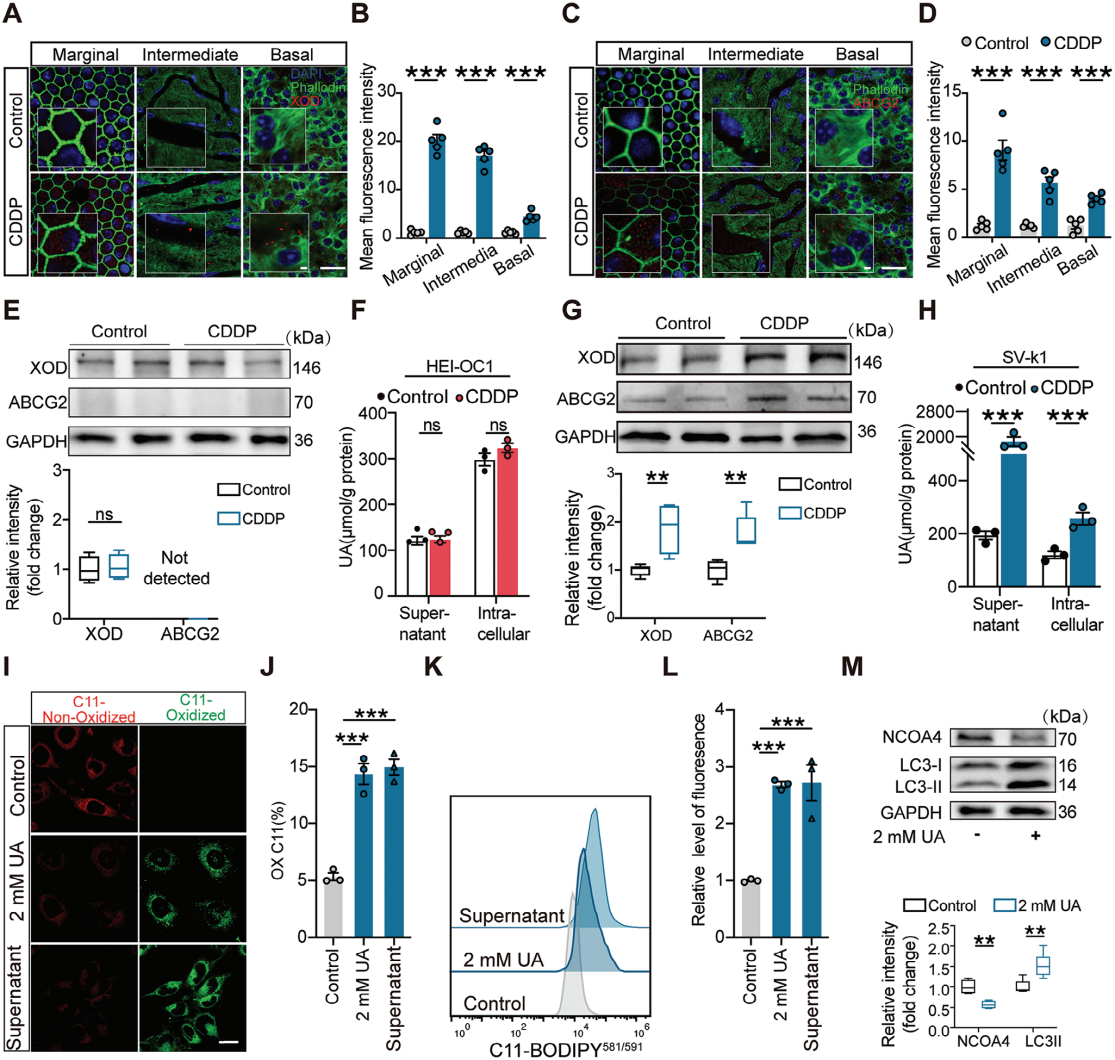
CDDP induces a notable release of uric acid from stria vascularis cells and subsequently triggers autophagy‐dependent ferroptosis of hair cells. A,C) Whole‐mount phalloidin staining (green) was used to visualize the three cell layers of the SV after CDDP administration. Costaining was performed with XOD or *ABCG2* antibodies (red). B,D) Quantification of XOD fluorescence intensity from (A) and *ABCG2* fluorescence intensity from (C), with *n* = 5 for each group. E) Western blot analysis of XOD and *ABCG2* protein levels in SV‐k1 cells after CDDP treatment (*n* = 3). F) Detection of supernatant and intracellular UA content in SV‐k1 cells after CDDP treatment for 24 h (*n* = 3). G) Western blot analysis of XOD and *ABCG2* protein levels in HEI‐OC1 cells after CDDP treatment (*n* = 3). H) Detection of supernatant and intracellular UA content in HEI‐OC1 cells after CDDP treatment for 24 h (*n* = 3). I,J) C11‐BODIPY581/591 immunofluorescence staining to detect lipid ROS in HEI‐OC1 cells: Supernatants from SV‐k1 cells treated with CDDP for 24 h were collected and cocultured with HEI‐OC1 cells or stimulated with 2 mM UA. K,L) Measurement of lipid ROS by flow cytometry. M) Western blot was used to detect the expression levels of NCOA4 and LC3 with or without UA intervention, followed by densitometric analysis (*n* = 3). Data are expressed as means ± SEM. *p < 0.05, **p < 0.01, ***p < 0.001 by student's *t* test.

Costaining with *ABCG2* and XOD antibodies (red) revealed changes in the cell layers after drug treatment (**Figure** **6**A–D). To explore the impacts of UA disposition in the specific cell subpopulations on the hair cells, the stria vascularis cell line SV‐k1 and hair cell line HEI‐OC1 were used for further study. After CDDP treatment, the protein levels of XOD and the UA transporter *ABCG2* were significantly upregulated in SV‐k1 cells (Figure 6G), consistent with changes observed in cochlear tissue of CDDP‐treated mice. By contrast, XOD levels in HEI‐OC1 cells did not change significantly after CDDP treatment, and the expression of the UA transporter *ABCG2* was undetectable (Figure 6E). Additionally, UA levels in both the intracellular environment and supernatants of HEI‐OC1 cells remained largely unchanged following CDDP administration (Figure 6F). However, both XOD and *ABCG2* expressions in SV‐k1 cells were significantly increased after CDDP treatment (Figure 6G). And the UA concentration in the supernatants of SV‐k1 cells increased tenfold, reaching 2 mm after CDDP treatment (Figure 6H). Therefore, we hypothesize that the abnormally high release of UA from stria vascularis cells may be transported through the endolymph fluid to trigger hair cell death. To further investigate how elevated UA levels cause HEI‐OC1 cell death, we analyzed iron content and lipid peroxidation markers in the serum, cochlea, and kidneys of CDDP‐treated mice. The results, as suggested by Kyoto Encyclopedia of Genes and Genomes (KEGG) enrichment analysis, indicated that ferroptosis plays a role in the observed tissue damage (Figure S6A–E, Supporting Information). Increasing evidence indicates that ferroptosis may be associated with CDDP‐ or neomycin‐induced ototoxicity, and recent studies have shown that autophagy‐dependent ferroptosis plays a key role in CDDP‐induced hearing loss.^[^ ^37^, ^38^, ^39^, ^40^ ^]^ However, the relationship between hyperuricemia and autophagy‐dependent ferroptosis in hair cells remains unclear. To investigate this, we first detected HEI‐OC1 intracellular lipid peroxidation levels using C11‐BODIPY 581/591 immunofluorescence staining combined with flow cytometry analysis (Figure 6I–L). The results showed that lipid peroxidation in HEI‐OC1 cells could be enhanced either by direct stimulation with an abnormally high UA concentration of 2 mm or by coincubation with supernatants from SV‐k1 cells after CDDP administration. Western blot analysis revealed significant changes in the ferritinophagy biomarkers LC3II and NCOA4 in hair cells after high UA exposure. Specifically, LC3II expression was upregulated and NCOA4 levels were downregulated, tentatively validating the activation of ferritinophagy during UA‐induced injury (Figure 6M).

### 2.7 Blockade of Excess Uric Acid Release from the Stria Vascularis Reverses CDDP‐Induced Hair Cell Damage

In the mammalian auditory system, the function of hair cell mechanotransduction depends on the intracochlear potential generated by the SV. The highly specialized capillary network within the SV, known as the blood–labyrinth barrier, controls the exchange between blood and cochlear endolymph. This barrier protects the inner ear from blood toxicants and plays a crucial role in maintaining cochlear homeostasis, which is essential for auditory function.^[^ ^41^, ^42^, ^43^, ^44^ ^]^

**Figure 7:**
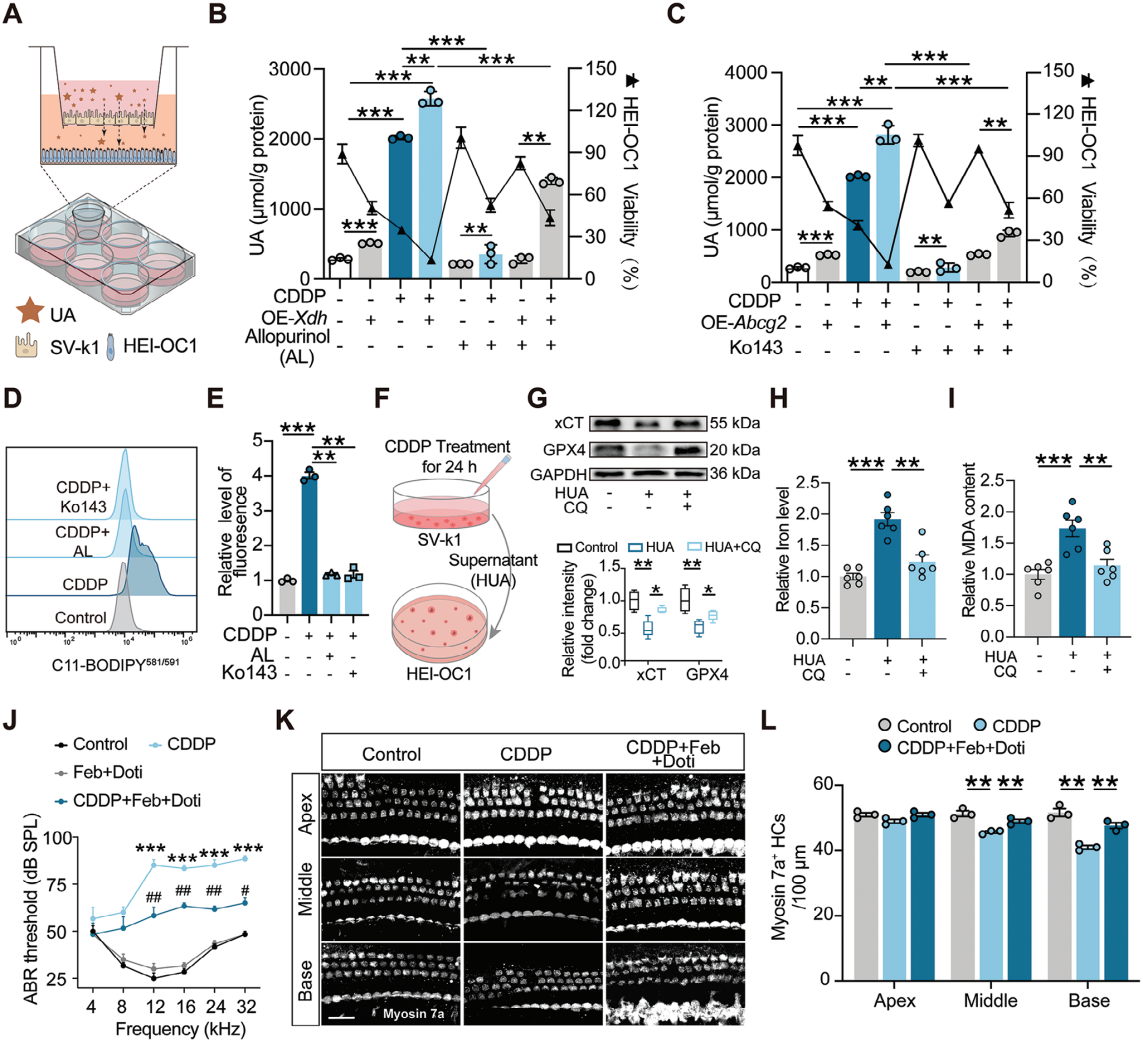
Blockade of excess uric acid release from the stria vascularis reverses CDDP‐induced hair cell damage and hearing dysfunction. A) Schematic illustrating the coculture of SV‐k1 cells with HEI‐OC1 cells. B,C) UA assay and cell viability in HEI‐OC1 cells from the indicated groups (*n* = 3). D,E) Detection of lipid ROS in HEI‐OC1 cells from the studied groups using flow cytometry. F) After 24 h of CDDP treatment to SV‐k1 cells, supernatants were collected and incubated with HEI‐OC1 cells, with or without CQ treatment, for subsequent assays. G) Western blot analysis of xCT and GPX4 levels in HEI‐OC1 cells, followed by quantification through grayscale analysis. H,I) Quantification of iron (H) and MDA (I) levels in each cell group. J) ABR measurements from the indicated groups (*n* = 3). ^#^ *p* < 0.05, ^##^ *p* < 0.01, versus CDDP group. K) Representative confocal images of cochlear hair cells immunostained for Myosin 7a (gray) in the indicated groups. Scale bar, 20 µm. L) Quantification of Myosin 7a‐positive hair cells in the studied groups (*n* = 3). Data are presented as means ± SEM. Statistical analysis: Two‐way ANOVA for (B, C); One‐way ANOVA for (E, G, H, I, J, L). *p < 0.05, **p < 0.01, ***p < 0.001. CQ, chloroquine; AL, allopurinol; Doti, dotinurad; Feb, febuxostat.

To investigate whether modulation of XOD and *ABCG2* in stria vascularis cells would promote hair cell ferroptosis by affecting perilymphatic UA levels, we used a SV‐k1–HEI‐OC1 coculture system to mimic the crosstalk between stria vascularis cells and hair cells (**Figure** **7**A). Allopurinol, which inhibits XOD by competing for its active site, and Ko143, which inhibits *ABCG2* by competitively inhibiting ATPase activity and altering its conformation, were utilized. SV‐k1 cells in the upper insert of the Transwell were treated with either XOD (or *ABCG2*) overexpression or inhibitor under a CDDP‐induced injury model. Cell viability of HEI‐OC1 cells in the lower compartment and UA levels were assayed. Quantitative assay results showed that SV‐k1 cells treated with CDDP significantly increased UA content in HEI‐OC1 cells in the lower compartment, leading to decreased cell viability. When *Xdh* or *ABCG2* were overexpressed separately, UA levels increased further, and cell viability decreased more. However, using inhibitors of the two genes partially blocked the elevated UA and rescued cell viability (Figure 7B,C). Further validation using four groups—control, CDDP, CDDP + allopurinol (AL), and CDDP + Ko143—showed that elevated lipid reactive oxygen species (ROS) in HEI‐OC1 cells after CDDP treatment was effectively reduced by AL or Ko143, suggesting that targeted interventions against XOD and *ABCG2* in the SV significantly influence ferroptosis in hair cells (Figure 7D,E and Figure S7I,J, Supporting Information). Additionally, incubating HEI‐OC1 cells with the supernatant of SV‐k1 cells treated with CDDP for 24 h showed that high uric acid (HUA) levels promoted ferroptosis in HEI‐OC1 cells (Figure 7F). This included iron accumulation, malondialdehyde (MDA) generation, GSH depletion, and downregulation of ferroptosis markers GPX4 and SLC7A11. Adding chloroquine to block autophagic flux significantly ameliorated these effects, indicating that autophagy blockade also reduced HUA‐induced iron accumulation and restored redox capacity in HEI‐OC1 cells (Figure 7G–I and Figure S7K (Supporting Information)). These findings suggest that drug‐induced abundant UA released from stria vascularis cells causes hair cell death through autophagy‐dependent ferritinophagy, and modulating UA metabolism and transport in stria vascularis cells may mitigate drug‐induced ototoxicity.

In addition, several clinical retrospective analyses have shown that the combination of UA excretion drugs with xanthine oxidase inhibitors effectively reduces serum uric acid (sUA) levels.^[^ ^45^, ^46^ ^]^ Based on these findings, we treated CDDP‐induced mice with febuxostat, one of the three FDA‐approved urate‐lowering drugs,^[^ ^47^ ^]^ in combination with Dotinurad, a novel UA reabsorption inhibitor (Figure S8A, Supporting Information).^[^ ^48^ ^]^ The results demonstrated that this combination therapy significantly reduced sUA levels without impairing liver function (Figure S8B–D, Supporting Information). Furthermore, measurements of Scr and blood BUN indicated significant renal protection. The mid‐to‐high frequency ABR threshold also showed marked improvement (Figure S8E,F, Supporting Information). Immunofluorescence staining of the hair cell marker Myosin VIIa revealed a substantial reduction in drug‐induced hair cell loss (Figure 7J–L). Taken together, these findings suggest that the simultaneous reduction of peripheral sUA levels during CDDP therapy effectively protects both kidney function and hearing, providing clinically relevant insights into mitigating nephrotoxicity and ototoxicity.

## 3 Discussion

**Figure 8:**
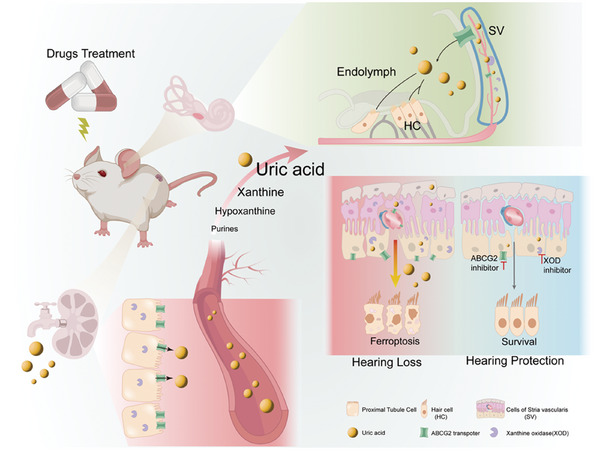
A schematic illustration of the role of elevated uric acid levels in drug‐induced ototoxicity and nephrotoxicity. Drug administration initially induces renal damage, leading to impaired excretion and subsequent accumulation of the toxic metabolite uric acid in the peripheral blood, which then infiltrates the cochlea. Xanthine oxidase and the uric acid transporter *ABCG2* are key regulators of uric acid metabolism and excretion, both of which undergo significant pathological changes in renal tubular epithelial cells and cochlear stria vascularis cells. Within the cochlea, targeted inhibition of specific genes in the stria vascularis cells effectively reduces uric acid levels, thereby preventing its accumulation in the endolymph and alleviating autophagy‐dependent ferroptosis in cochlear hair cells.

To date, the common material basis and precise mechanisms of drug‐induced renal dysfunction and hearing loss have not been well elucidated. In this study, we found that UA, an end product of purine metabolism, gradually accumulates in the serum of mice during disease progression and is specifically elevated in kidney and inner ear tissues, as revealed by untargeted metabolomics. Kidney damage following the initial cycle of CDDP administration disrupted systemic UA homeostasis, resulting in elevated systemic UA concentrations. Notably, UA accumulation in the cochlea was observed at a later time point. Through ex vivo and in vivo experiments, we verified that higher‐than‐physiological levels of UA can not only cause ear and kidney tissue damage alone but also aggravate drug‐induced ear and kidney toxicity and exacerbate sensorineural deafness such as noise exposure. Mechanistically, as shown in **Figure** **8**, drug‐induced renal dysfunction prevents the excretion of the nephrotoxic substance UA, which accumulates in peripheral blood and is transported to the inner ear. Ototoxic drugs upregulate XOD and *ABCG2* in the stria vascularis cells, facilitating further transport of UA from the blood–labyrinth barrier to the endolymph, thereby activating autophagy‐dependent ferroptosis in hair cells.

Hyperuricemia has become a worldwide metabolic disease and is considered the “fourth cause” of hypertension, hyperglycemia, and hyperlipidemia.^[^ ^49^ ^]^ Previous studies have shown that metabolic diseases negatively affect hearing. For example, hypertension accelerates hearing loss, possibly due to vascular damage;^[^ ^50^, ^51^ ^]^ hyperlipidemia deteriorates cochlear morphology and function, with statins showing protective effects against hearing loss.^[^ ^52^, ^53^ ^]^ As early as 1987, Axelsson and Sigroth proposed diabetes‐related hearing loss, primarily affecting nerve fibers or auditory sensory cells in the inner ear.^[^ ^54^, ^55^, ^56^ ^]^ Thus, abnormal metabolic processes are often inextricably linked to hearing loss. In this study, similar to other metabolic disorders, UA levels were significantly positively correlated with hearing dysfunction. XOD and *ABCG2*, which control UA bioprocesses, were significantly upregulated after ototoxic drug administration. Transport of UA by *ABCG2* and overproduction of UA by upregulation of XOD are common mechanisms in pathological disorders with high UA. We validated for the first time the localization of *ABCG2* and XOD in the mouse inner ear, primarily in the SV structures in the cochlea.

Hearing loss induced by CDDP or aminoglycosides is usually bilateral, starting at high frequencies and extending to lower frequencies with prolonged treatment.^[^ ^7^ ^]^ Hair cells in the organ of Corti have long been recognized as the primary targets of CDDP and aminoglycoside antibiotics, but they are not the only targets. Aminoglycosides affect the SV,^[^ ^57^ ^]^ with CDDP preferentially accumulating in the stria, leading to impaired stria function.^[^ ^58^ ^]^ The SV is responsible for ion exchange to maintain high potentials in the endolymph and low potentials in the ectolymph, creating potential gaps critical for sensory hair cell transduction.^[^ ^9^ ^]^ Therefore, drug‐induced hair cell death may result from impaired maintenance of endolymph by the SV. In this study, ototoxic drugs disrupted the integrity of the blood–labyrinth barrier, allowing toxic UA to enter the endolymph while overexpressing XOD and *ABCG2* in the stria vascularis cells, causing more UA accumulation in the hair cells and reducing hair cell survival.

Increasing research focuses on the impact of ferroptosis on hearing problems, and autophagy‐dependent ferroptosis is involved in the pathogenesis of hyperuricemic nephropathy.^[^ ^59^ ^]^ Ferroptosis is a newly identified type of regulatory cell death mediated mainly by iron‐dependent lipid peroxidation.^[^ ^60^ ^]^ Both CDDP and hyperuricemic acid exposure promote ROS activity and increase ROS production.^[^ ^59^, ^61^ ^]^ We hypothesized that drug‐induced high levels of UA may contribute to the progression of hearing loss by modulating ferroptosis in hair cells. The occurrence of ferroptosis was verified by measuring iron, GSH content, MDA levels, and levels of the ferritin GPX4 and SLC7A11. It was shown that intracellular iron overload is an autophagy‐dependent process. This specific autophagic pathway, called ferritinophagy, is characterized by the binding of ferritin to lysosomes. In this process, lysosomes selectively degrade ferritin to free iron under the guidance of NCOA4. We verified that hyperuricemia activated autophagy‐dependent ferritinophagy by using the autophagy inhibitor chloroquine in combination with Bodipy C11 staining and detecting the iron‐autophagy protein NCOA4 and the autophagy marker LC3II. When XOD or *ABCG2* was inhibited in the SV, ferritinophagy was attenuated, and the inhibition of hair cell activity by hyperuricemia was reversed.

In the future, further investigation should be concentrated on the impact of regulating UA transport in the kidneys on the cochlea, exploring systemic nephro–cochlea interactions. This will involve targeting UA transporters and metabolic enzymes using advanced techniques such as adeno‐associated‐virus‐mediated gene delivery or CRISPR‐based genome editing to assess how modulating UA processing in the kidneys influences cochlear toxicity. Additionally, the inclusion of samples from patients with hyperuricemia or gout will enable us to validate the clinical relevance of our findings and gain a deeper understanding of how chronically elevated UA levels affect hearing function. Ultimately, we should identify novel biomarkers for the early detection of toxicity and to develop targeted strategies to prevent or mitigate urate‐induced cochlear damage. The real‐time monitoring of UA levels in patients undergoing CDDP therapy will allow us to conduct dynamic adjustments to CDDP dosing to prevent exacerbated hearing loss.

In conclusion, this study identified the important role of UA in drug‐induced ototoxicity and nephrotoxicity through metabolomics. It is demonstrated for the first time that elevated UA levels can directly result in hearing loss through autophagy‐dependent ferroptosis. UA supplementation or inhibition of its excretion exacerbated hearing loss and hair cell damage. Targeted interventions against XOD and *ABCG2* in stria vascularis cells reduced UA release and subsequently attenuated ferroptosis in hair cells. This study revealed a metabolic link between the inner ear and kidney in drug‐induced ototoxicity and nephrotoxicity, highlighting UA modulation as a potential therapeutic strategy for hearing loss. This study also suggests that preventive measures should be implemented to guard against irreversible hearing loss in patients with hyperuricemia.

## 4 Experimental Section

### Chemicals

The following chemical reagents were used in this study: cisplatin (S31072, Yuan Ye, Shanghai, China), neomycin (HY‐B0470, MedChemExpress, NJ, USA), potassium oxonate (HY‐17511, MedChemExpress), chloroquine (HY‐17589A, MedChemExpress), allopurinol (HY‐101397, MedChemExpress), dotinurad (Fuji Yakuhin Co., Tokyo, Japan), febuxostat (HY‐14268, MedChemExpress), uric acid (HY‐B2130, MedChemExpress), and Ko143 (HY‐10010, MedChemExpress).

### Animal Procedures

All animal procedures were conducted in accordance with the National Institutes of Health Guide for the Care and Use of Laboratory Animals and were approved by the Ethics Committee on Animal Experimentation of China Pharmaceutical University (SYXK 2021‐0011, Nanjing, China).

### Drug Treatment

Male C57BL/6J mice (8 weeks old, weighing 20–22 g) were obtained from Beijing Vital River Laboratory Animal Technology Co. (Beijing, China). CDDP administration was performed as previously described.^[^ ^9^, ^13^ ^]^ Briefly, CDDP‐treated mice received three rounds of once‐daily intraperitoneal injections (3.5 mg kg^−1^) for 4 days, followed by 10 days of recovery, for a total duration of 42 days. Saline‐treated mice received a parallel regimen of saline administered at an equivalent volume to their CDDP‐treated counterparts. Male BALB/c mice (3 weeks old, weighing 14–16 g) were obtained from Beijing Vital River Laboratory Animal Technology Co. (Beijing, China). Neomycin was administered as follows: neomycin‐treated mice were injected intraperitoneally with 100 mg kg^−1^ once daily for 14 days. Control mice received the same volume of saline as the neomycin‐treated mice.

### Hyperuricemic Mice and Drug Administration

Male BALB/c mice (3 weeks old, weighing 14–16 g) were obtained from Beijing Vital River Laboratory Animal Technology Co. (Beijing, China). The animals were randomly assigned into four groups, with 6 mice per group: for the increased UA production group, mice received a daily intraperitoneal injection of 250 mg kg^−1^ UA, as reported previously.^[^ ^62^ ^]^ For the decreased UA excretion group, mice were orally administered 250 mg kg^−1^ potassium oxonate by gavage once daily, as mentioned previously.^[^ ^49^ ^]^ For the combination modeling group, mice received a UA intraperitoneal injection 30 min after daily gavage with potassium oxonate. For the control group, mice were administered normal saline. These mice were used for further experiments, including hearing and kidney function tests, after 4 days of continuous administration.

### Noise Exposure

Male BALB/c mice were exposed to white noise at 110 dB for 2 h in a soundproof chamber, either in a high uric acid environment or not. The sound was generated by a Tucker Davies Technologies (TDT) system and amplified by high fidelity. After two weeks, ABR tests and immunofluorescence analyses were performed to examine the auditory function of the mice.

### ABR Measurement

The ABR threshold was commonly used to assess hearing function. Mice were anesthetized with sodium pentobarbital (100 mg kg^−1^) via intraperitoneal injection. Active electrodes were inserted at the skull base, with a reference electrode positioned below the tested ear and a ground electrode near the tail. ABR thresholds of the mice were recorded at six frequencies (4, 8, 12, 16, 24, and 32 kHz) using the BioSigRZ software (TDT, Gainesville, FL, USA) and a Tucker Davis Technology System III. Sound pressure level (dB) was measured in 5 dB increments from 10 to 90 dB. The hearing threshold was defined as the lowest sound pressure level that elicited a detectable auditory response.

### Immunohistochemistry

After the mice were sacrificed by cervical dislocation, the inner ear and kidney were collected. For the kidney samples, a portion of the kidney was fixed with 4% paraformaldehyde (PFA) in phosphate‐buffered saline (PBS) at 4 °C overnight and then dehydrated in 20% and 30% sucrose–phosphate‐buffered saline solution successively at 4 °C until it sank to the bottom of the tube. The dehydrated tissues were embedded in O.C.T compound (4583, Sakura Finetek, CA, USA) and stored at −80 °C overnight. Frozen sections were then sliced into 10 µm sections using a cryostat (Leica CM 1850, Wetzlar, Germany) for further immunostaining. For the inner ear samples, the cochleae were fixed in 4% PFA at 4 °C overnight and then decalcified in 0.5 m ethylenediaminetetraacetic acid (EDTA) for at least 5 h. The cochleae were dissected into pieces or incubated in 15% and 30% glucose, followed by O.C.T embedding and frozen sectioning. The sections were then blocked with 0.3% Triton X‐100 and 8% donkey serum for 1 h at room temperature before adding primary antibodies. Tissue sections were labeled with rabbit anti‐*ABCG2* (1:1000, 27286‐1‐AP, Proteintech, IL, USA) and mouse anti‐XOD (1:500, sc‐398548, Santa Cruz, TX, USA) overnight at 4 °C. After three rinses with PBS, 1:500 secondary antibodies were applied for 1 h. All Alexa Fluor secondary antibodies were from Thermo Scientific: goat anti‐mouse (IgG2a) Alexa Fluor 647 (A‐21241) and donkey anti‐rabbit Alexa Fluor 555 (A‐31572). DAPI (D3571, Thermo Scientific, USA) was added at 5 mg mL^−1^ in secondary antibodies at a 1:1000 dilution to stain nuclei. After washing with PBS, the sections were covered with an antifade mounting medium, sealed with nail polish, and observed under a laser scanning confocal microscope (Olympus Corporation, Tokyo, Japan).

### Kidney Histology

For histological analysis, a portion of the renal cortex was fixed with 4% PFA in PBS, embedded in paraffin wax, and then sliced into 5 µm sections for hematoxylin and eosin staining, periodic acid–Schiff staining, and Masson's trichrome staining. The stained sections were imaged using an upright microscope (Olympus Corporation, Tokyo, Japan).

### Immunofluorescence

The mouse cochlear temporal bone was rapidly dissected in cold PBS and then fixed in 4% PFA. For whole‐mount staining, the stria vascularis was dissected out and fixed in 4% PFA for 30 min on ice. For the organ of Corti, cochleae were transferred into PBS and microdissected under a microscope following 4% PFA fixation and 0.5 m EDTA decalcification. The mouse cochlear basement membrane and stria vascularis were dissected using a microscope and adhered to a round glass slide with Cell‐Tak (354240, Corning, NY, USA). The cochlear tissue was immersed in PBST (1% Triton X‐100 in PBS) solution for 15 min and then incubated in blocking solution (10% goat serum in PBS) at room temperature for 1 h. The cochlear tissue was then incubated in PBS containing primary antibody overnight at 4 °C. The next day, the tissue was washed 3 times for 5 min each in PBST solution and then incubated in PBS solution containing secondary antibody at room temperature for 1 h. The secondary antibodies were removed, and the samples were incubated with DAPI or phalloidin (1:2000, A12379, Thermo Scientific) for 30 min at room temperature. Finally, the samples were covered with an antifade mounting medium after washing in PBST. Cochlear samples were visualized using a confocal microscope (Olympus Corporation, Tokyo, Japan). The following antibodies were used: *Xdh* (1:1000, 55156‐1‐AP, Proteintech), *ABCG2* (1:1000, 27286‐1‐AP, Proteintech), ZO‐1 (1:200, MA3‐39100‐A647, Thermo Scientific), Myo7a (1:200, 25‐6790, Proteus Biosciences, CA, USA), Myo7a (1:100, sc‐74516, Santa Cruz), Alexa Fluor 555‐donkey anti‐rabbit (1:2000, A‐31572, Thermo Scientific), and Alexa Fluor 647‐goat anti‐mouse (1:2000, A‐21241, Thermo Scientific).

### Cochlear Explants

P2 C57BL/6J mice were decapitated and cleaned with 75% alcohol. The inner ears were isolated and transferred to precooled sterile HBSS. The cochleae were then microdissected under a microscope to remove capsules and modiolus. The sensory epithelium was separated from the ligaments and seeded on Cell‐Tak‐coated coverslips to be cultured in Dulbecco's modified Eagle medium (DMEM)‐F12 medium supplemented with 1% N2 supplement (A1370701, Thermo Scientific) and 2% B27 solution (17504044, Thermo Scientific) overnight at 37 °C with 5% CO_2_. They were then randomly divided into groups for treatment with different compounds. Next, the culture medium was removed, and the samples were fixed with 4% PFA in PBS for further immunostaining.

### Renal Function Analysis

Commercial kits were used to measure the levels of serum creatinine (E‐BC‐K188‐M, Elabscience, Wuhan, China) and blood urea nitrogen (E‐BC‐K183‐M, Elabscience) in serum or tissue lysates, following the manufacturer's instructions.

### Liver Function Analysis

Commercial kits were used to measure serum aspartate aminotransferase levels (C010‐3‐2, Jiancheng) and blood alanine aminotransferase levels (C009‐3‐2, Jiancheng) in serum, following the manufacturer's instructions.

### Cell Culture

The HEI‐OC1 cells were cultured in DMEM (Gibco, USA) supplemented with 10% FBS at 37 °C in a humidified incubator with 5% CO_2_. The mouse proximal renal tubular cell line (TCMK‐1) was purchased from the American Type Culture Collection (Rockville, MD, USA). TCMK‐1 cells were cultured in EMEM (Gibco, USA) supplemented with 10% FBS at 37 °C in an incubator with 5% CO_2_. SV cells (SV‐k1) were purchased from Bluefbio in China. SV‐k1 cells were cultured in high‐glucose medium (SH30022.01, Hyclone, UT, USA) supplemented with 10% FBS at 37 °C in an incubator with 5% CO_2_.

### Plasmids and Transfection

SV‐k1 cells were seeded in 24‐well plates at a density of 1 × 10^5^ cells per well, and the complete medium was replaced with serum‐free medium before transfection. Using the Lipofectamine 3000 (L3000001, Thermo Scientific) liposome method, the cells were transfected with an empty vector plasmid (vector), *Xdh* overexpression plasmid, and *ABCG2* overexpression plasmid. All plasmids were purchased from GenePharma (Shanghai, China). After 24 h of transfection, the efficacy of the various treatments was determined by quantitative real‐time polymerase chain reaction (PCR) and Western blotting.

### CCK‐8 Cell Viability Assay

A CCK‐8 (C0043, Beyotime, Shanghai, China) was used to determine cell viability. Cells (1 × 10^4^) were cultured in a 96‐well plate and pretreated with the relevant conditional medium or compound. After treatment, cells were incubated with CCK‐8 for 1 h at 37 °C, and the absorbance value was measured at 450 nm.

### Quantitative Real‐Time PCR (qPCR)

According to the manufacturer's instructions, total RNA was extracted from mouse tissue or cells using Trizol reagent (R401, Vazyme, Nanjing, China). cDNA was synthesized using a reverse transcription kit (R323, Vazyme). Subsequently, qPCR was performed with a mixture containing 10 µL SYBR Green qPCR Mix (Q311, Vazyme), 0.4 µL Primer F, 0.4 µL Primer R, 1 µL Template cDNA, and 8.2 µL ddH_2_O. Each sample was run in triplicate, and the relative expression was calculated using the 2−ΔΔCT method. The primer sequences used were as follows: Glut9 (F: 5′‐TTGCTTTAGCTTCCCTGATGTG‐3′; R: 5′‐GAGAGGTTGTACCCGTAGAGG‐3′), *Xdh* (F: 5′‐ATGACGAGGACAACGGTAGAT‐3′; R: 5′‐TCATACTTGGAGATCATCACGGT‐3′), Ada (F: 5′‐GTCACCCCTGATGACGTTGTG‐3′; R: 5′‐CAGAATGGACCGGACCTTGAT‐3′), Oat1 (F: 5′‐GGCACCTTGATTGGCTATGT‐3′; R: 5′‐CCACAGCATGGAGAGACAGA‐3′), Oat3 (F: 5′‐CGGAATAGCCAACCACAACT‐3′; R: 5′‐ATCACAGGTCCTCCAACCAg‐3′), Oat4 (F: 5′‐ATGGCTACTCTGTGCTCCTG‐3′; R: 5′‐GTCAGCAGAGATGGTGAGGA‐3′), Urat1 (F: 5′‐CGCTTCCGACAACCTCAATG‐3′; R: 5′‐CTTCTGCGCCCAAACCTATCT‐3′), Prps1 (F: 5′‐ACTTATCCCAGAAAATCGCTGAC‐3′; R: 5′‐CCACACCCACTTTGAACAATGTA‐3′), *ABCG2* (F: 5′‐CACTGACCCTTCCATCCTCTTC‐3′; R: 5′‐GCCCTGTTTAGACATCCTTTTCA‐3′), and β‐actin (F: 5′‐ACGGCCAGGTCATCACTATTG‐3′; R: 5′‐AGGGGCCGGACTCATCGTA‐3′).

### Western Blot

For cell lysis, medium‐strength RIPA lysis buffer (P0013B, Beyotime), protease inhibitors mixture (ST507, Beyotime), and protein phosphatase inhibitor (KGP602, KeyGen Biotech, Nanjing, China) were added to the cells, and the cells were placed on ice for 30 min. The samples were subsequently centrifuged at 16 000 *g* for 10 min at 4 °C, and the supernatant was collected, supplemented with sodium dodecyl sulfate (SDS) denaturing buffer, boiled for 10 min, and subjected to SDS‐PAGE and western blotting analysis after transferring onto polyvinylidene fluoride membranes (IPVH00010, Millipore, MA, USA). The membranes were blocked with 5% skim milk (P0216, Beyotime) in TBST for 2 h and then incubated overnight at 4 °C with primary antibodies diluted in 5% BSA (A8020, Solarbio, Beijing, China) prepared in TBST. Subsequently, membranes were washed 3 times (10 min each) with TBST, followed by incubation with secondary antibodies (diluted in 5% skim milk) for 1 h at room temperature. ECL (E412‐01, Vazyme) substrate was added to visualize the signal. For protein extraction from other tissues of sacrificed mice, the inner ear, liver, and kidney were quickly removed, placed in a mixture of medium‐strength RIPA lysis buffer, protease inhibitor, and protein phosphatase inhibitor, thoroughly ground and lysed on ice for 30 min, and centrifuged at 16 000 *g* for 10 min at 4 °C. The supernatant was collected and subjected to SDS‐PAGE and western blotting analysis as described above. The following primary antibodies were used: *Xdh* (1:1000, 55156‐1‐AP, Proteintech), *ABCG2* (1:1000, 27286‐1‐AP, Proteintech), NCOA4 (1:1000, DF4255, Affinity Biosciences, OH, USA), LC3B (1:1000, 3868, Cell Signaling Technology, MA, USA), xCT (1:2000, DF12509, Affinity Biosciences), GPX4 (1:5000, ab125066, Abcam, Cambridge, UK), GAPDH (1:10 000, 60004‐1, Proteintech), β‐actin (1:1000, 4967, Cell Signaling Technology).

### Enzyme‐Linked Immunosorbent Assay (ELISA)

Serum, tissue, or cell samples were used for ELISA tests following the manufacturers' protocols. Tissue samples were weighed, added to precooled 10% physiological saline, and centrifuged at 4 °C and 4000 rpm for 15 min. The protein concentration was quantified using a BCA assay (P0012, Beyotime). The activity of XOD was detected in the tissues and serum using an assay kit (A002‐1‐1, Jiancheng, Nanjing, China). The activity of ADA was detected in the tissues and serum using an assay kit (R22207, Yuan Ye). The cellular GSH levels of HEI‐OC1 cells were assessed using a corresponding assay kit (A006, Jiancheng). The cellular free iron levels of HEI‐OC1 cells were measured using an iron colorimetric assay kit (E1042, Applygen, Beijing, China). All kits were tested on a microplate absorbance reader (Bio‐Tek Instruments, Winooski, VT, USA).

### Uric Acid Assay

To determine UA levels, a UA Assay Kit (C012‐2‐1, Jiancheng) was used. UA levels in serum were measured according to the manufacturer's instructions. For tissue and cell samples, UA levels were calculated using a standard curve generated from the absorbance values of the UA standards provided in the kit. Tissue samples were weighed, 250 µL of ultrapure water was added, and the mixture was centrifuged at 4 °C and 18 000 rpm for 5 min. Cell samples were collected, homogenized with PBS, and centrifuged at 18 000 rpm for 5 min at 4 °C. The supernatants from the tissue and cell samples were collected, and the protein content was quantified by BCA analysis to correct the UA concentration.

### Measurement of Lipid Peroxidation

HEI‐OC1 cells were collected and fragmented by ultrasonic fragmentation. The supernatants were collected by centrifugation, and the protein concentration was quantified using a BCA assay. The intracellular MDA level was measured with a Lipid Peroxidation MDA Assay Kit (A003, Jiancheng) according to the manufacturer's instructions. Lipid ROS was detected using a BODIPY 581/591 C11 probe (D3861, Thermo Scientific). Briefly, cells were incubated with 2 µm BODIPY 581/591 C11 at 37 °C for 30 min, and fluorescence was detected on a flow cytometer at the fluorescein isothiocyanate green channel (LSR Fortessa, BD, NJ, USA). A minimum of 10 000 live cells were collected, and data were analyzed using FlowJo software. Additionally, after staining for 30 min in the dark, the cells were washed 3 times and immediately observed using an Olympus microscope. Oxidized BODIPY and reduced BODIPY were observed at excitation/emission wavelengths of 488/510 and 581/591 nm, respectively.

### Metabolomics Analysis

At 40 days following the CDDP model, serum, kidney, and cochlea samples were collected from both control and CDDP groups (*n* = 6–7 per group). l‐Glutamine‐^13^C_5_ (HY‐N0390S1, MedChemExpress) was used as an internal standard. For sample collection and preparation, 50 µL of serum was added to 400 µL of methanol solution containing 1.5 µg mL^−1^ of the internal standard. For kidney tissue, 20 mg of mouse kidney tissue was weighed and added to 800 µL of 80% methanol solution containing 1.5 µg mL^−1^ of the internal standard. Cochlear samples were prepared with an internal standard to achieve a final concentration of 1.5 µg mL^−1^. The protein was precipitated, shaken for 5 min, and then allowed to stand at 4 °C for 1 h. The supernatant was centrifuged at 20 000 *g* for 10 min. The solvent was evaporated under reduced pressure using vacuum centrifugation and a rotary evaporator, then completely evaporated and redissolved with 100 µL of ultrapure water. The samples were then centrifuged at 4 °C and 18 000 rpm for 10 min, and the supernatant was transferred to vials for LC–MS analysis. Pooled QC samples were pretreated following the same procedure and processed after every ten actual serum samples.

A Waters HPLC system (Waters, Milford, MA, USA) coupled with a 5500 triple‐quad mass spectrometer (AB SCIEX, Los Angeles, CA, USA) was used for separation and metabolic analysis. Briefly, the injection volume was 20 µL, the column temperature was set to 30 °C, and the flow rate was 0.4 mL min^−1^. The mobile phase consisted of aqueous phase A (950 mL of 5 mm ammonium acetate, pH adjusted to 9.0 with 10% ammonia, plus 50 mL of acetonitrile) and organic phase B (acetonitrile). Separation was carried out using gradient elution with the following program: 0–3 min 85% B, 3–6 min 85–30% B, 6–15 min 30–2% B, 15–18 min 2% B, 18–19 min 2–85% B, and 19–26 min 85% B. Negative ion mode scan analysis was performed with a scanning range for TOF MS scan of *m*/*z* 50–1000 and an ion scanning range of *m*/*z* 50–900. Data were analyzed using Analyst TF 1.7, MultiQuant 3.0 software (AB SCIEX), and R packages (“fmsb,” “VennDiagram,” “ComplexHeatmap”). Partial least squares discriminant analysis and metabolomics pathway analysis of the differential compounds were conducted using MetaboAnalyst (http://www.metaboanalyst.ca/, accessed on 30 June 2024).

### Single‐Nuclear RNA Sequencing

Three FVB mice were sacrificed at P20. Cochleae and kidneys collected from different mice were combined as single samples, respectively. After homogenization and centrifugation, the nuclei were partitioned into individual droplets with a barcode gel bead using the 10X Chromium instrument (10X Genomics, Pleasanton, CA). The Seurat package (version 4.3.1) was used for dimension reduction, cell clustering, and differential gene expression analyses. UMAP was used for the final dimension reduction and visualization. Marker genes were identified by comparing the mean expression of each gene in one cell type against the mean expression in all other cell types using the function FindAllMarkers with the parameter method = MAST in the Seurat package. Top marker genes were selected based on the adjusted *p*‐value and log_2_ fold change within each cluster, and cell clusters were determined based on previously known cell type marker expression. CellChat was used for cell communication analysis. The clusterProfiler package was used to perform KEGG analysis of DEGs between different clusters. Furthermore, visualization of gene expression by scatter plots, dot plots, and violin plots was implemented through the functions “FeaturePlot(),” “DotPlot(),” and “VlnPlot()” in the Seurat package.

### Statistical Analysis

Statistical analyses were conducted using GraphPad Prism 9.0 software. Data were expressed as mean ± standard error of the mean (SEM). To test for statistical significance, the Student's *t*‐test was used to compare two different groups. Comparisons between more than two groups were analyzed using two‐way analysis of variance (ANOVA) with Dunnett's test or one‐way ANOVA with Tukey's test. Statistical significance was defined as a *p*‐value less than 0.05, with significance levels indicated as *p* < 0.05 (*), *p* < 0.01 (**), and *p* < 0.001 (***).

## Conflict of Interest

The authors declare no conflict of interest.

## Author Contributions

S.G., C.C., Y.W., and K.S. contributed equally to this work. S.G., C.C., Y.W., and K.S. performed the majority of experiments, analyzed the data, and drafted the paper. D.Z., B.C., and X.W. performed partial experiments and acquired the data. R.C., G.W., and F.Z. designed the study, supervised the experiments, revised the paper. All authors read and approved the final paper.

## Supporting information



Supporting Information

## Data Availability

The data that support the findings of this study are available from the corresponding author upon reasonable request.
