# Supplementary material for: Metabolomic and Cellular Mechanisms of Drug‐Induced Ototoxicity and Nephrotoxicity: Therapeutic Implications of Uric Acid Modulation
Source: Adv Sci (Weinh). 2025 Mar 5;12(16):2415041. doi: 10.1002/advs.202415041 (PMC12021111; doi:10.1002/advs.202415041)
Supplement: Supplementary file 1 — Supporting Information [file ADVS-12-2415041-s001.docx]

**
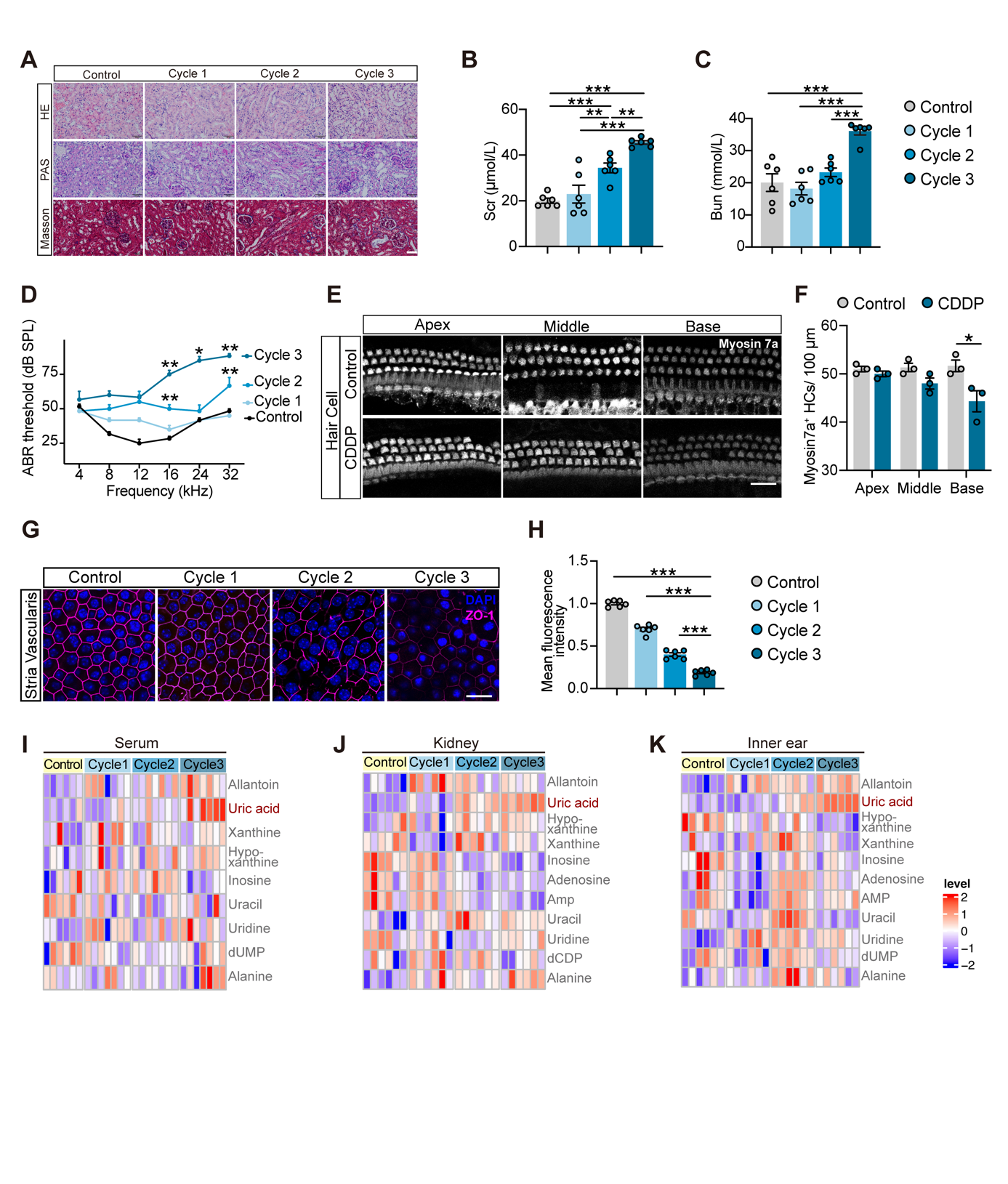
**

**Figure. S1.** Metabolomics analysis in the cisplatin-induced ototoxicity and nephrotoxicity model. (A) H&E, PAS, and Masson staining of mice renal tissues from the studied groups, scale bar: 50 µm. (B-C) Scr and BUN levels in mice kidneys from the studied groups over three cycles (n=6). (D) ABR measurements after three cycles of CDDP treatment from the studied groups (n=3). (E) Representative confocal images of hair cells were stained with Myosin7a (grey) in mice cochlea after CDDP treatment from the studied groups, scale bar: 20 µm. (F) Myosin7a^+^ OHC quantification in mice after CDDP treatment from the studied groups (n=3). (G) Representative confocal microscope images demonstrating the compact linear structure of ZO-1 (magenta) on the plasma membrane in the stria vascularis, scale bar: 20 µm. (H) Mean fluorescence intensity in the stria vascularis in (G), as assessed with ImageJ software (n=6). (I-K) Heatmap showing changes in levels of metabolites involved in purine and pyrimidine metabolism in serum, kidney, and cochlea after three cycles of CDDP treatment from the studied groups. Data are expressed as means ± SEM. Statistical analysis: two-way ANOVA for (D); one-way ANOVA for (B, C, F, H). **p* < 0.05, ***p* < 0.01, ****p* < 0.001. PAS, Periodic Acid-Schiff; H&E, Hematoxylin and Eosin; Masson, Masson's Trichrome; Scr, Serum creatinine; BUN, Blood urea nitrogen; ZO-1, Zonula-occludens 1.


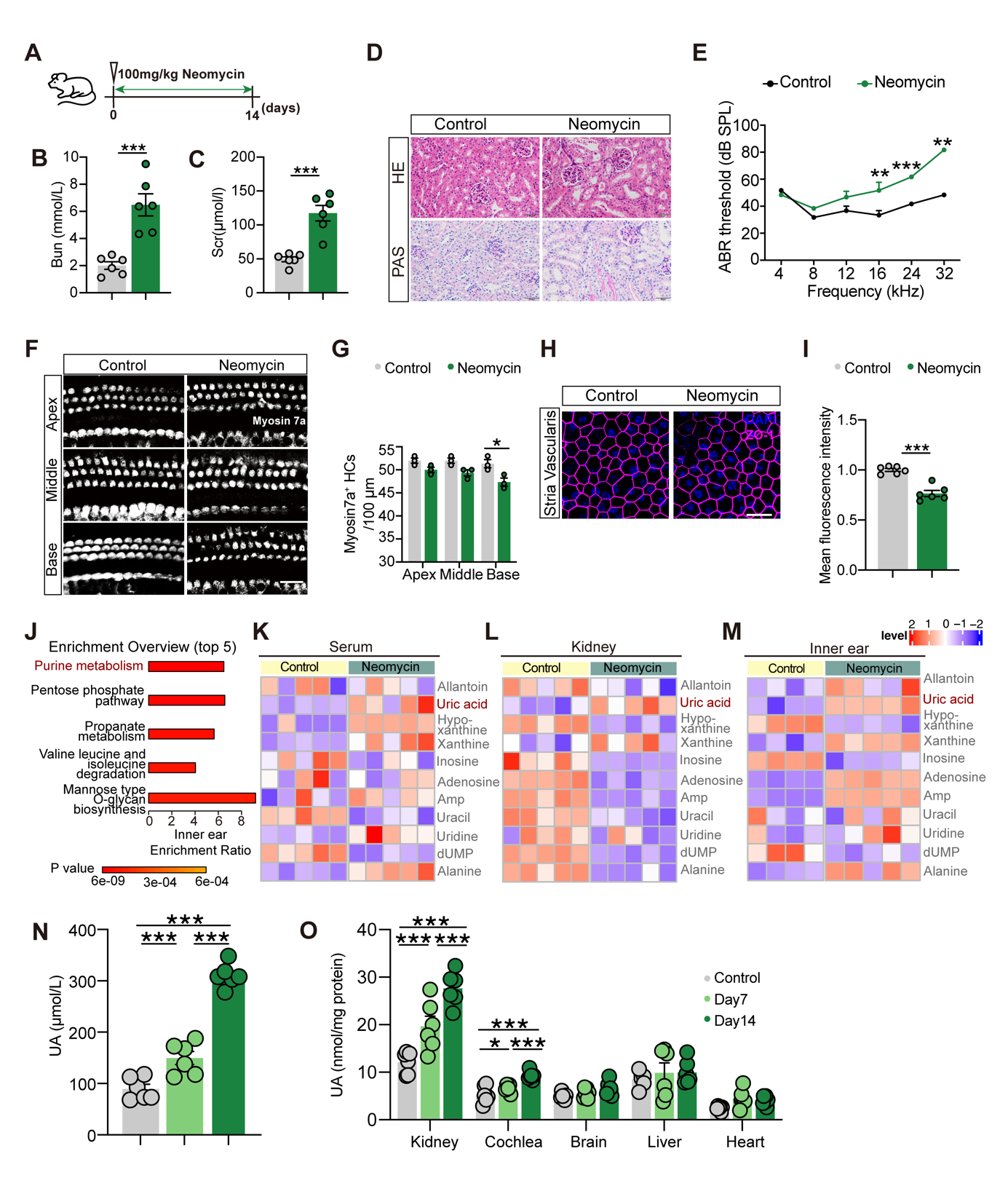


**Figure. S2.** Metabolomics analysis in the neomycin-induced ototoxicity and nephrotoxicity model. (A) Schematic diagram of drug administration. (B-C) Scr and BUN levels in mice kidneys from the studied groups after 14 consecutive days of neomycin administration. (D) H&E and PAS staining of mice renal tissues from the studied groups, scale bar: 50 µm. (E) ABR measurements after 14 consecutive days of neomycin treatment from the studied groups (n=3). (F) Representative confocal images of hair cells were stained with Myosin7a (grey) in mice cochlea after neomycin treatment from the studied groups, scale bar: 20 µm. (G) Quantification of Myosin7a^+^ HCs in mice after neomycin treatment from the studied groups (n=3). (H) Representative confocal microscope images demonstrating the compact linear structure of ZO-1 (magenta) on the plasma membrane in the stria vascularis, scale bar: 20 µm. (I) Mean fluorescence intensity in the stria vascularis in (J), assessed with ImageJ software (n=6). (J) KEGG pathway enrichment analysis in the inner ear after neomycin treatment. (K-M) Heatmaps displaying changes in metabolites involved in purine and pyrimidine metabolism pathways in serum, kidney, and inner ear from the studied groups (n=4-5). (N-O) Quantitative detection of UA levels in serum and various tissues in mice after neomycin treatment. Data are expressed as means ± SEM. Statistical analysis: two-way ANOVA for (I); one-way ANOVA for (N,O); Student’s t test for (B, C, E, F, H). **p* < 0.05, ***p* < 0.01, ****p* < 0.001.


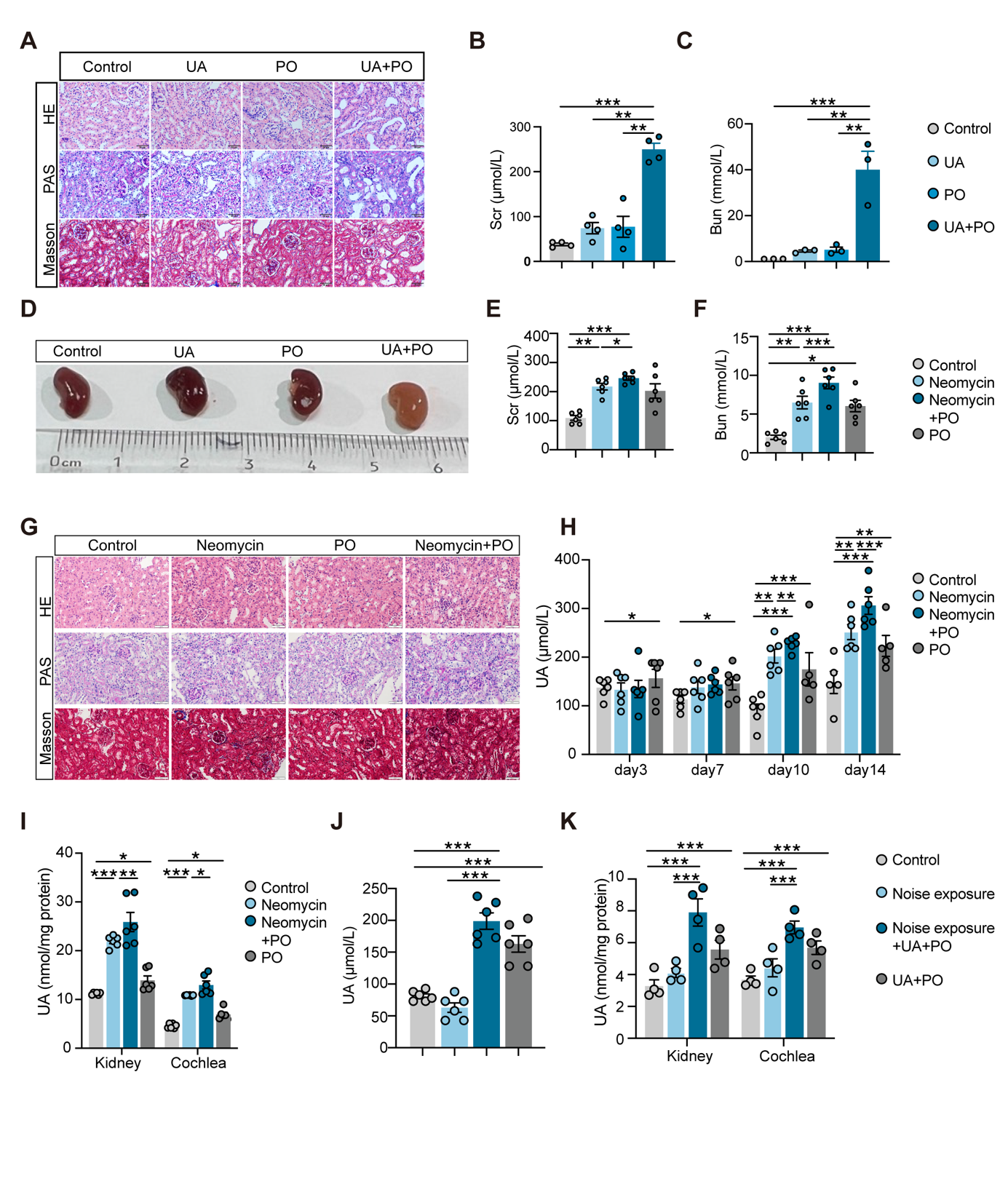


**Figure. S3.** Elevated uric acid levels exacerbate renal function damage and hearing loss. (A) H&E, PAS, and Masson staining of mice renal tissues from the studied groups, scale bar: 50 µm. (B-C) Scr and BUN levels in mice kidneys from the studied groups (n=4). (D) Gross appearance of whole kidneys from each group, scale bar: 1 mm. (E-F) Scr and BUN levels in mice kidneys from the studied groups (n=6). (G) H&E, PAS, and Masson staining of mice renal tissues from the studied groups, scale bar: 50 µm. (H) Quantification of serum UA levels in the study group on day 3, day 7, day 10, and day 14 (n=6). (I) Quantitative detection of UA levels in kidneys and cochleae from the studied groups (n=6). (J-K) Quantitative detection of UA levels in serum, kidneys, and cochleae from the studied groups (n=6). Data are expressed as means ± SEM. **p* < 0.05, ***p* < 0.01, ****p* < 0.001 by one-way ANOVA.

**
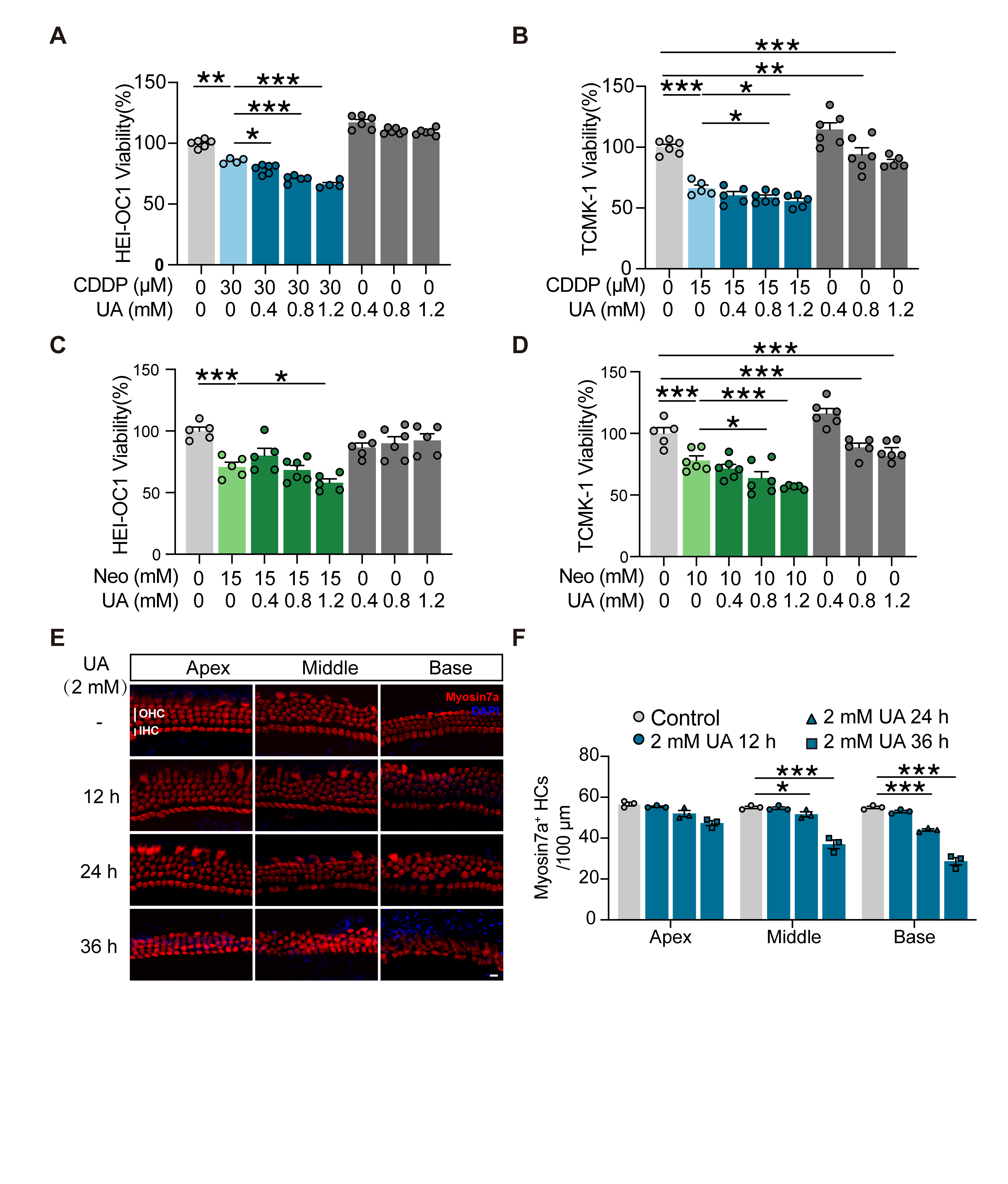
**

**Figure. S4.** Exogenous uric acid supplementation increased otorenal cytotoxicity induced by neomycin or CDDP. (A-B) Survival rates of HEI-OC1 and TCMK-1 cells treated with CDDP and UA, either individually or in combination, were assessed using the CCK-8 assay. (C-D) Survival rates of HEI-OC1 and TCMK-1 cells treated with neomycin and UA, either individually or in combination, were also assessed using the CCK-8 assay (n=5-6). (E-F) Immunofluorescence analysis of hair cells (HCs) at 0, 12, 24, and 36 hours following treatment with 2 mM UA. Myosin 7a-positive HCs appear in red, and nuclei are stained in blue. Quantification of HCs in each treatment group is shown (n = 3). Data are expressed as means ± SEM. **p* < 0.05, ***p* < 0.01, ****p* < 0.001 by one-way ANOVA.


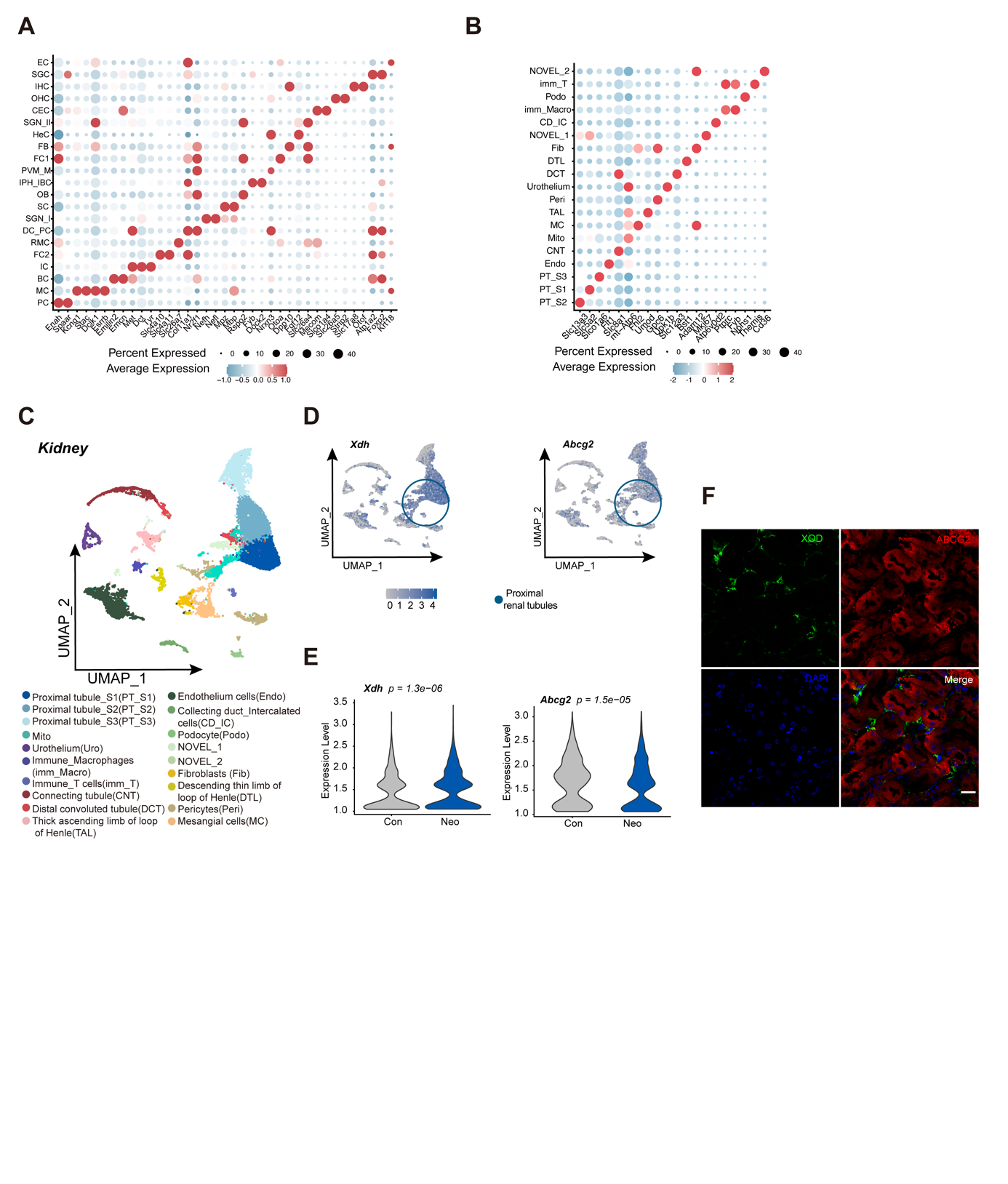


**Figure. S5.** Establishment of single-cell transcriptome landscape of mouse cochlea and kidney after neomycin administration.(A) Dot plot showing the expression of canonical cell-type-specific marker genes for diverse cochlear cell types. (B) Dot plot showing the expression of canonical cell-type-specific marker genes for diverse kidney cell types. (C) UMAP plot showing the distribution of different cell types in the kidney, with annotations as follows: PT, proximal tubule; Mito, mitochondria; Uro, urothelium; imm_Macro, immune macrophages; imm_T, immune T cells; CNT, connecting tubule; DCT, distal convoluted tubule; TAL, thick ascending limb of the loop of Henle; Endo, endothelial cells; CD_IC, collecting duct intercalated cells; PODO, podocyte; Novel_1; Novel_2; Fib, fibroblasts; DTL, descending thin limb of the loop of Henle; Peri, pericytes; MC, mesangial cells. (D) UMAP plot displaying Xdh and Abcg2 gene distribution in the kidney snRNA-seq dataset. (E) Violin plots of selected gene expression in proximal tubule cells after neomycin administration. (F) Representative immunohistochemical images of XOD (green) and ABCG2 (red) in mouse kidney tissues.


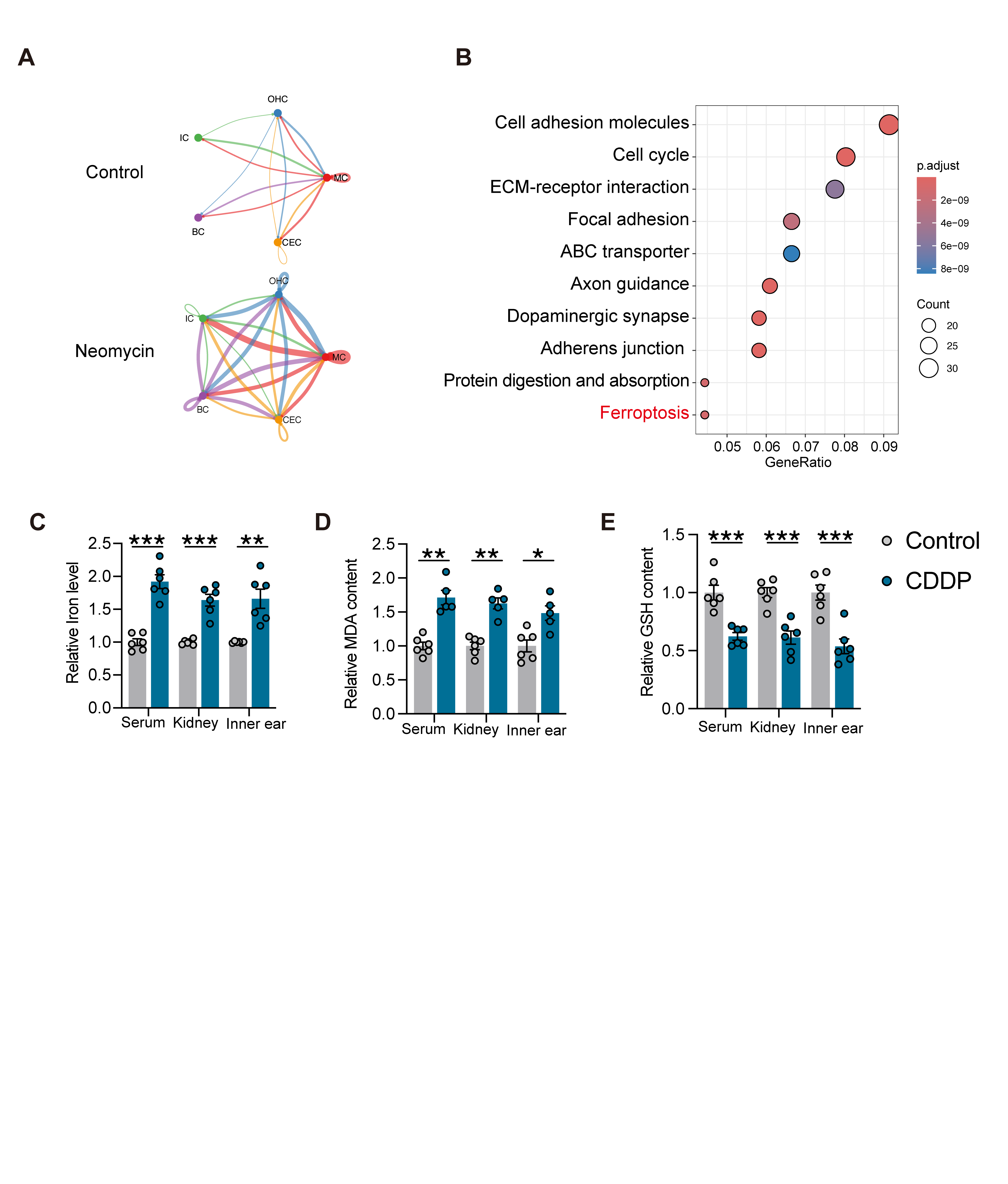


**Figure. S6.** CDDP induces elevated levels of uric acid produced by stria vascularis cells and subsequent autophagy-dependent ferroptosis of hair cells. (A) Circle plots displaying the differential number of interactions and interaction strengths in the cochlea between MC, IC, and BC of the stria vascularis subset, and OHC and IHC of the hair cell subset after neomycin treatment. (B) KEGG enrichment analysis of differentially expressed genes in the cochlea of control and neomycin-treated mice. (C–E) Quantitative analyses of iron (C), MDA (D), and GSH (E) levels in the serum, cochleae and kidneys following CDDP treatment (n = 6). Data are presented as mean ± SEM. **p* < 0.05, ***p* < 0.01, ****p* < 0.001 by one-way ANOVA.


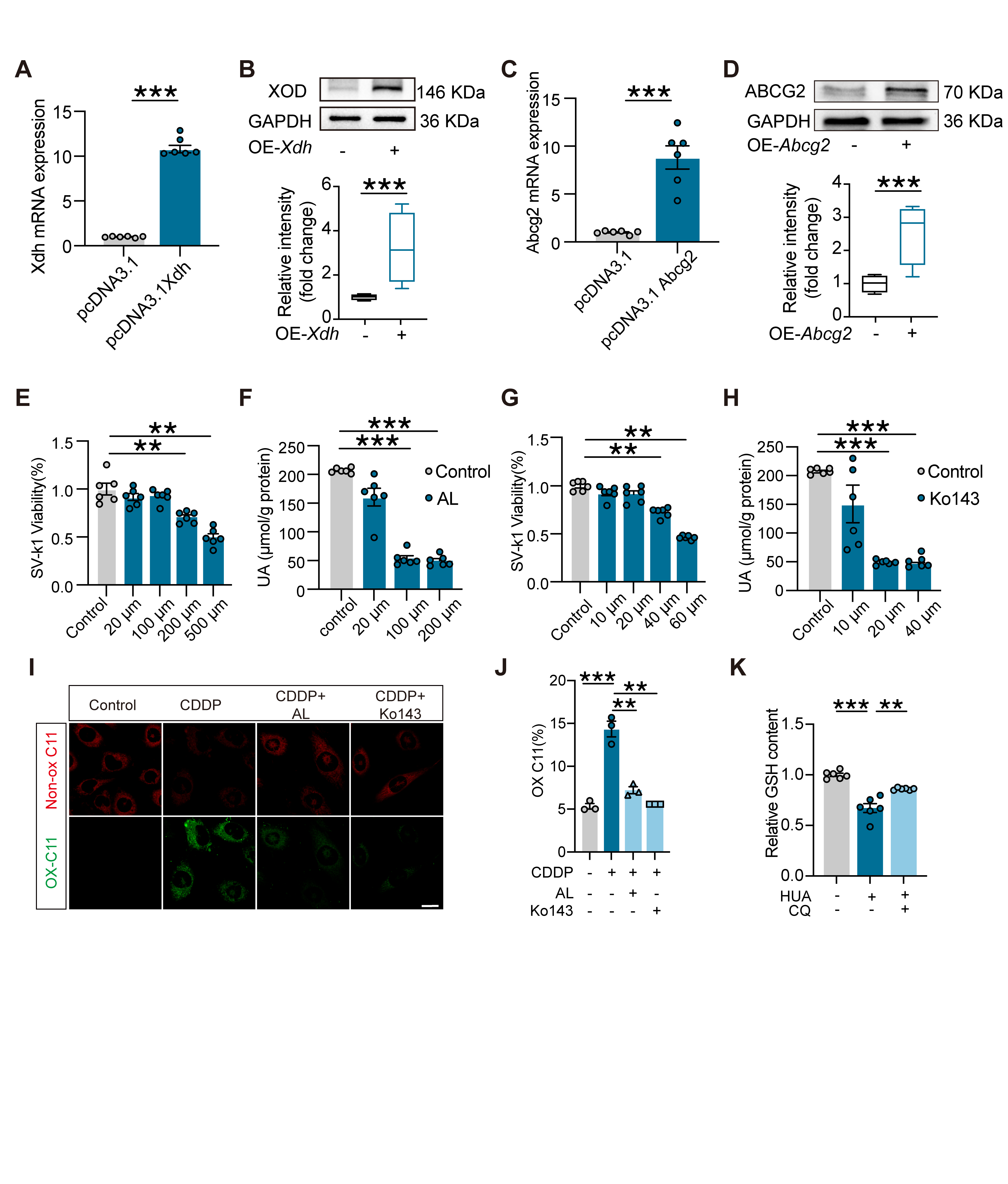


**Figure. S7.** Blockade of Excess Uric Acid Release from the Stria Vascularis Reverses CDDP-Induced Hair Cell Damage. (A, C) mRNA expression levels of XOD and ABCG2 in SV-k1 cells (n = 6). (B, D) Western blot analysis of XOD and ABCG2 protein levels (n = 3). (E, G) Cell viability of SV-k1 cells after 24 h of allopurinol or Ko143 treatment, respectively (n = 6). (F, H) UA content in the SV-k1 cell supernatant following 24 h of allopurinol or Ko143 treatment (n = 6). (I, J) Detection of lipid ROS in HEI-OC1 cells from the studied groups using immunofluorescence staining. (K) Quantification of GSH level in each cell group. Data are expressed as means ± SEM. **p* < 0.05, ***p* < 0.01, ****p* < 0.001 by student’s t test.


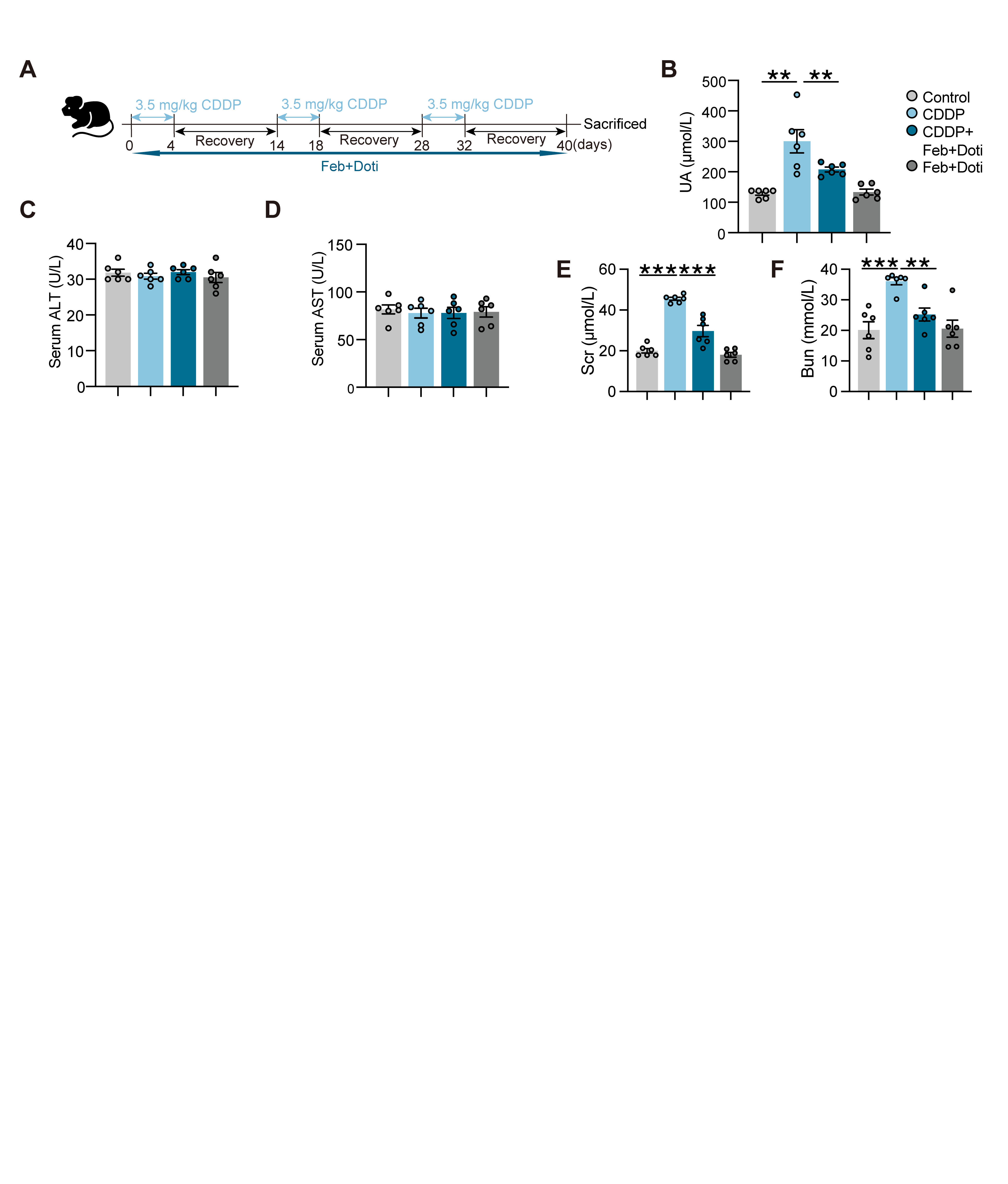


**Figure S8.** Blocking the CDDP-Induced Increase in Blood Uric Acid Effectively Preserves Renal and Hearing Function. (A) Schematic diagram of the drug administration protocol. (B) Quantification of serum UA levels in the indicated experimental groups (n = 6). (C-D) Measurement of serum aspartate aminotransferase (AST) and alanine aminotransferase (ALT) levels in each group (n = 6). (E-F) Scr and BUN levels in the kidneys of the indicated groups (n = 6). Data are expressed as means ± SEM. Statistical analysis: one-way ANOVA for (B, C, D, E, F). **p* < 0.05, ***p* < 0.01, ****p* < 0.001. Doti, Dotinurad; Feb, Febuxostat.
